# Supplementary material for: Characteristics and filtering of low-frequency artificial short deletion variations based on nanopore sequencing
Source: Gigascience. 2025 Mar 21;14:giaf018. doi: 10.1093/gigascience/giaf018 (PMC11927395; doi:10.1093/gigascience/giaf018)
Supplement: giaf018_Supplemental_File [file giaf018_supplemental_file.zip › Supplementary_Figure1-15.pdf]

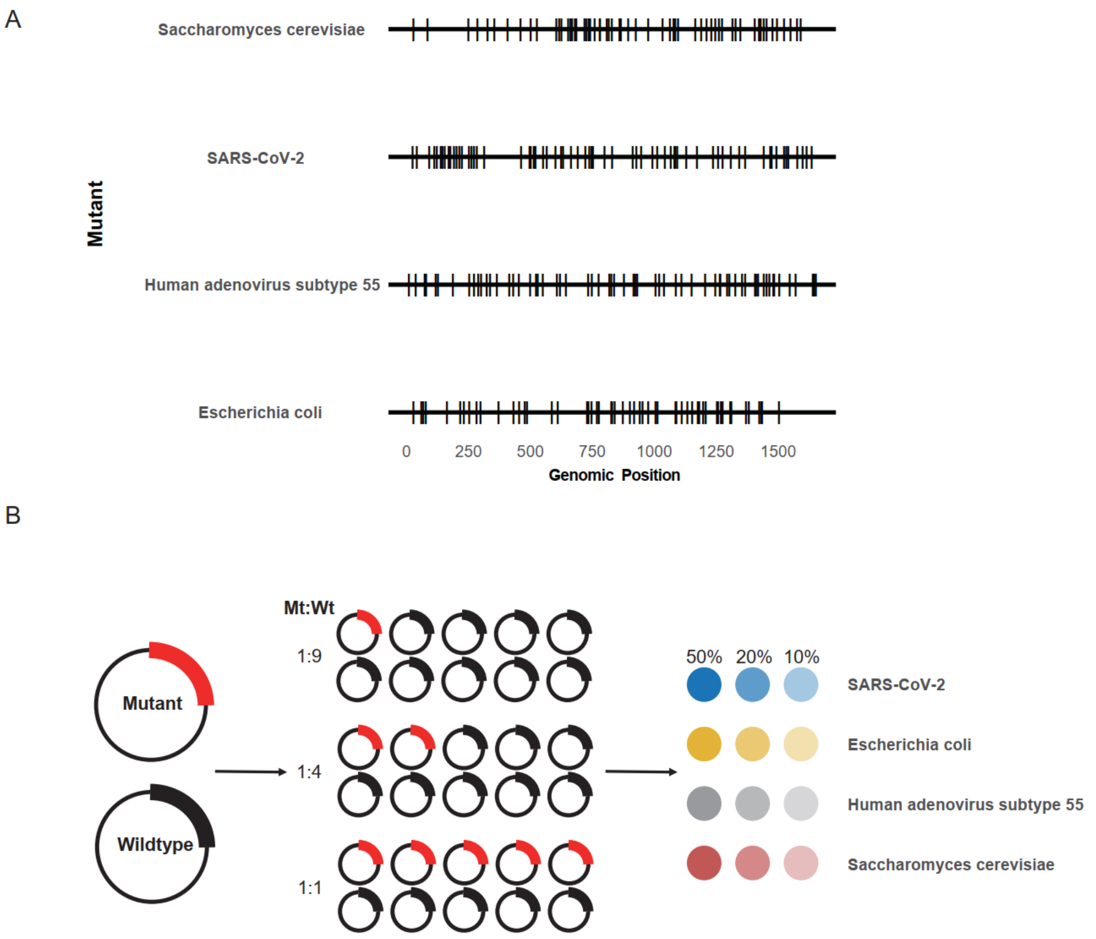

Supplementary Figure S1. The design of synthetic sequences and mixture samples. (A) The position of deletion variations of the mutated sequences are illustrated as vertical lines. (B) Scheme of mixing the wildtypes and mutants. The mutant and wildtype plasmids were mixed with 1:9, 1:4, and 1:1 ratios for each mixture.

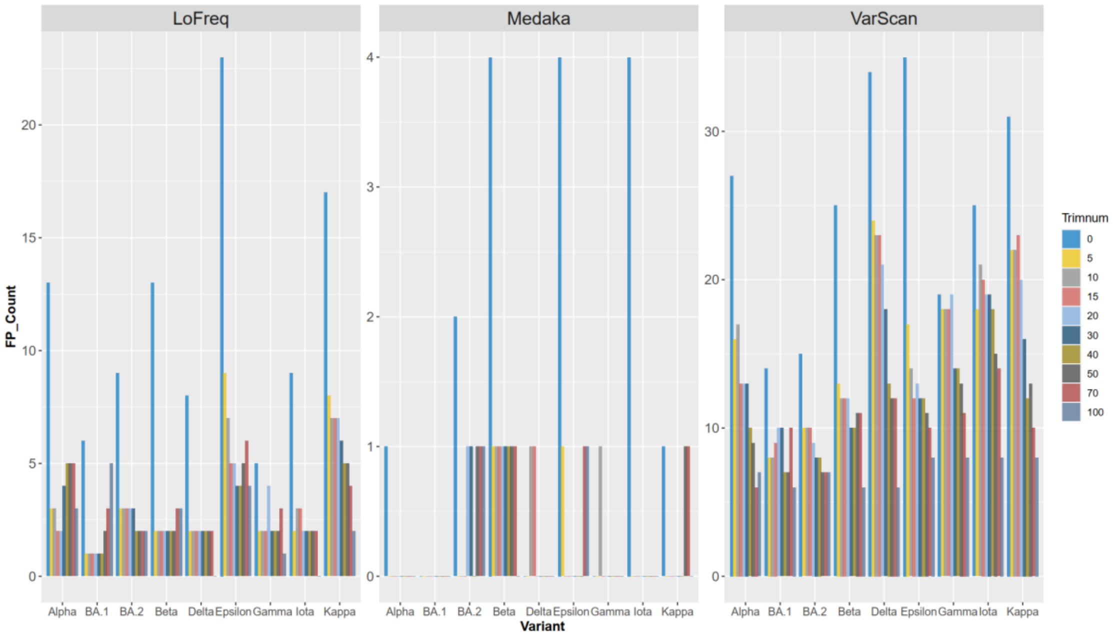

Supplementary Figure S2. The counts of artificial variations before and after trimming bases in different SARS-CoV-2 variants by three variant callers. Only variations with MuAF ≥0.2 were plotted. Trimnum means the number of trimmed bases.

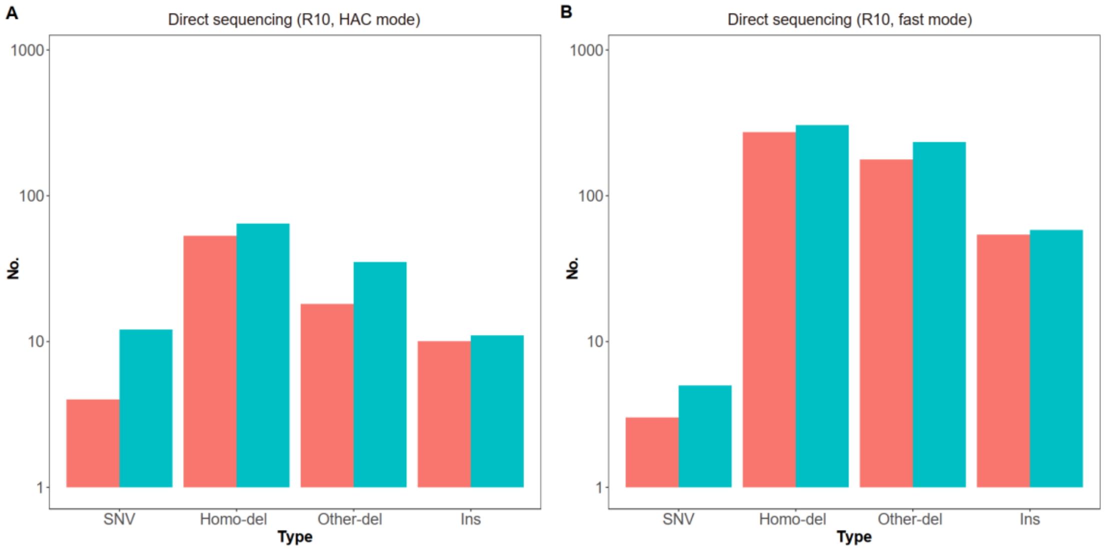

Supplementary Figure S3. The bar plots of four types of artificial variations before (red) and after (blue) trimming bases in R10 direct sequencing samples basecalled with the HAC model (A) and fast model (B). The y-axis was log10 transfromed.

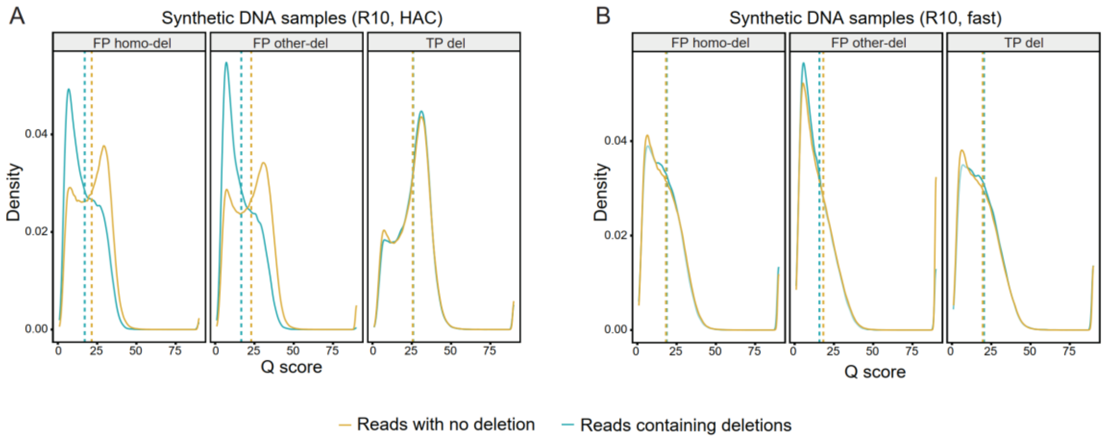

Supplementary Figure S4. The comparisons of Q scores between reads containing deletion variations and reads with no deletions in R10 direct sequencing data basecalled with the HAC model (A) and fast model (B). The dashed lines represented the mean values of Q scores.

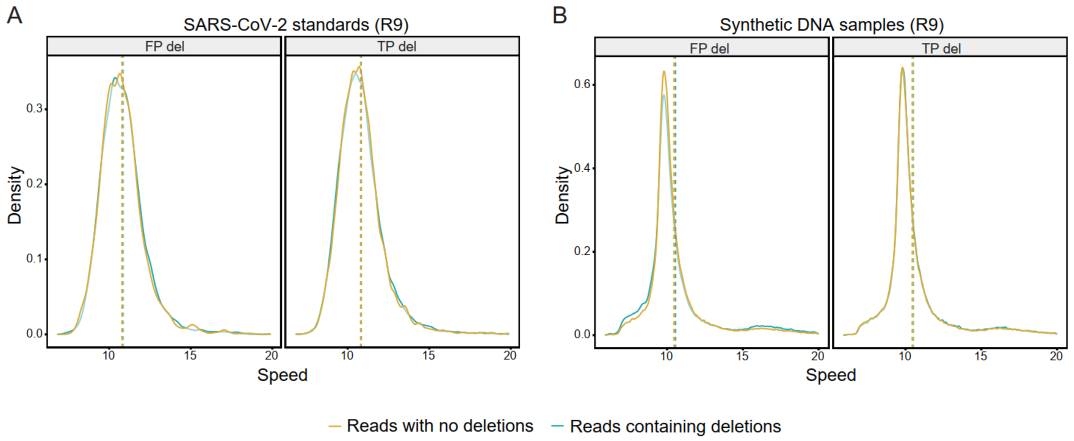

Supplementary Figure S5. The comparisons of sequencing speed between reads containing deletion variations and reads with no deletions in R9 WTA sequencing data (A) and R9 direct sequencing data (B) at the scale of the whole read. The dashed lines represented the mean values of speeds. Sequencing speed equals the division of the number of current measurements by the base number.

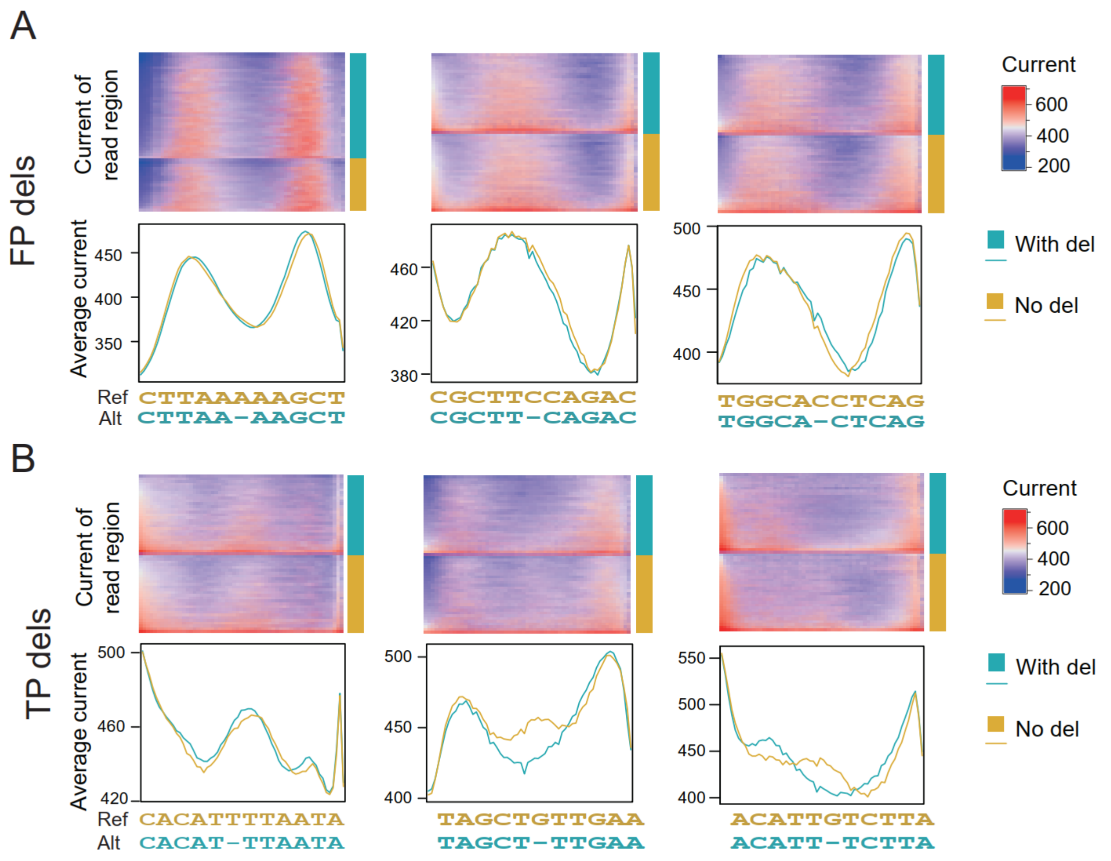

Supplementary Figure S6. The features of normalized current signals in R9 sequencing data. (A) Heatmaps of normalized current signals from reads with and without 1-base FP homo-del and 1-base FP other-del variations. The line plots represented each column's average normalized current measurements in the heatmap. The alternate alleles corresponding to deletions were displayed. (B) Heatmaps of normalized current signals from reads with and without 1-base TP homo-del and 1-base TP other-del variations.

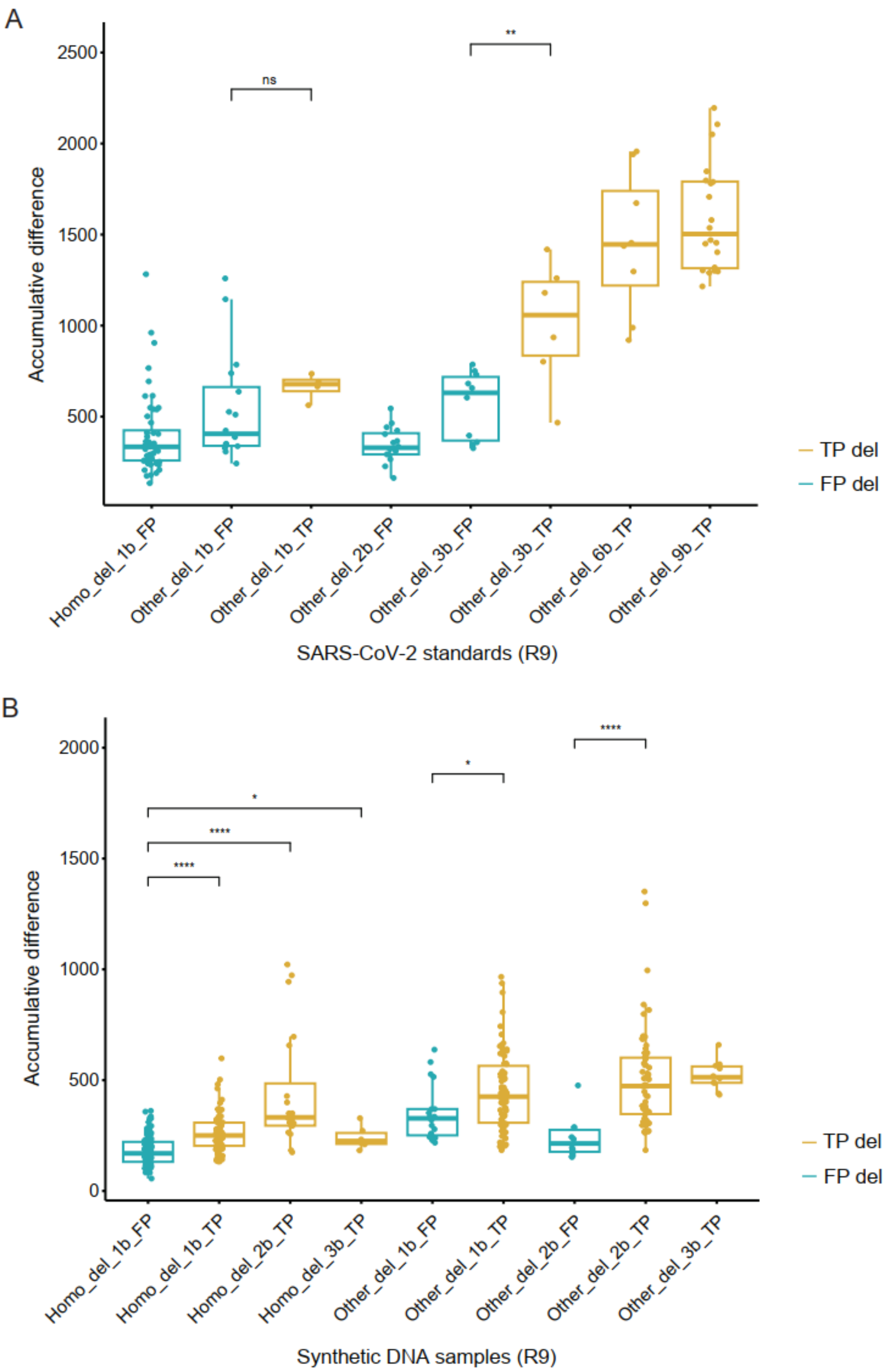

Supplementary Figure S7. The accumulative difference of average normalized current measurements from FP and TP deletions stratified by deletion length in R9 WTA sequencing data (A) and R9 direct sequencing data (B). Boxes represent the interquartile range (IQR) between the first and third quartiles (25th and 75th percentiles, respectively). Lines inside denote the median, and whiskers denote the most extreme values within 1.5 times IQR from the first and third quartiles. Outlier values are represented as points. ns: P > 0.05; *P ≤

0.05; **P ≤ 0.01; ****P ≤ 0.0001. 1b: 1-base; 2b: 2-bases; 3b: 3-bases; 6b: 6-bases; 9b: 9-bases.

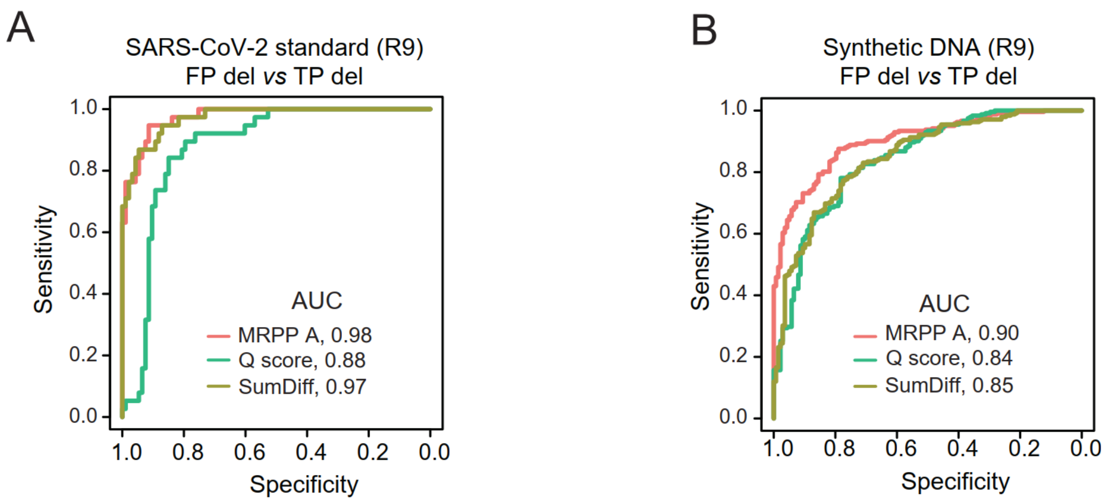

Supplementary Figure S8. Performance assessment of different indexes in distinguishing between FP and TP deletions in R9 sequencing data. (A) The ROCs of MRPP A, Q score, and accumulative difference in distinguishing between all FP and TP deletions in R9 WTA sequencing data. (B) The ROCs of MRPP A, Q score, and accumulative difference in distinguishing between all FP and TP deletions in R9 direct sequencing data.

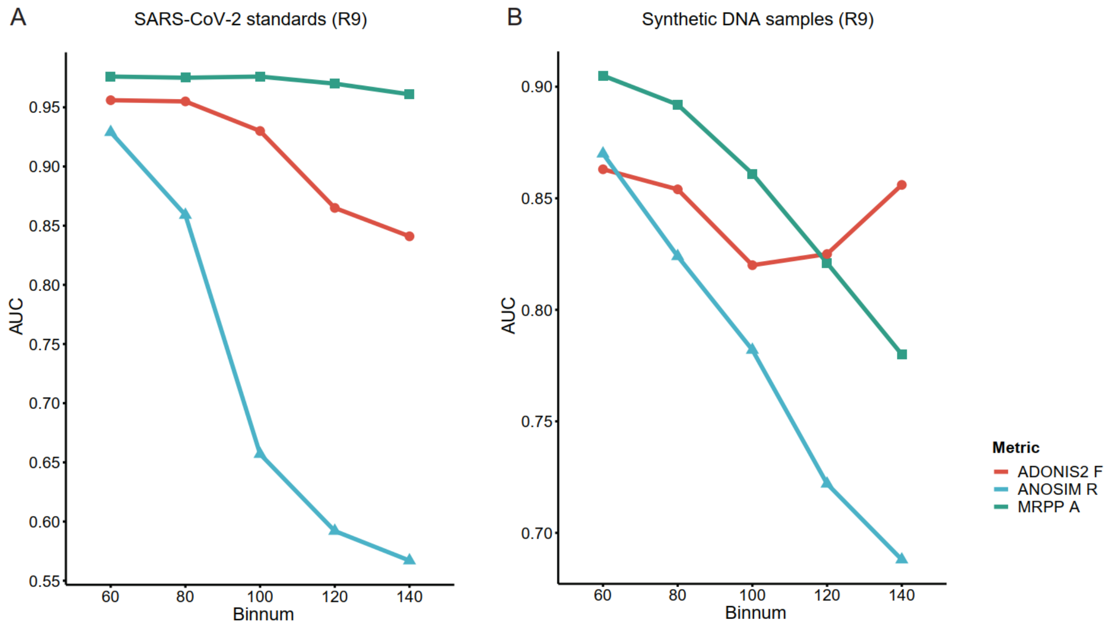

Supplementary Figure S9. The AUCs across different bin numbers of MRPP A, ANOSIM R, and ADONIS2 F in distinguishing between FP and TP deletions in R9 WTA sequencing data (A) and R9 direct sequencing data (B).

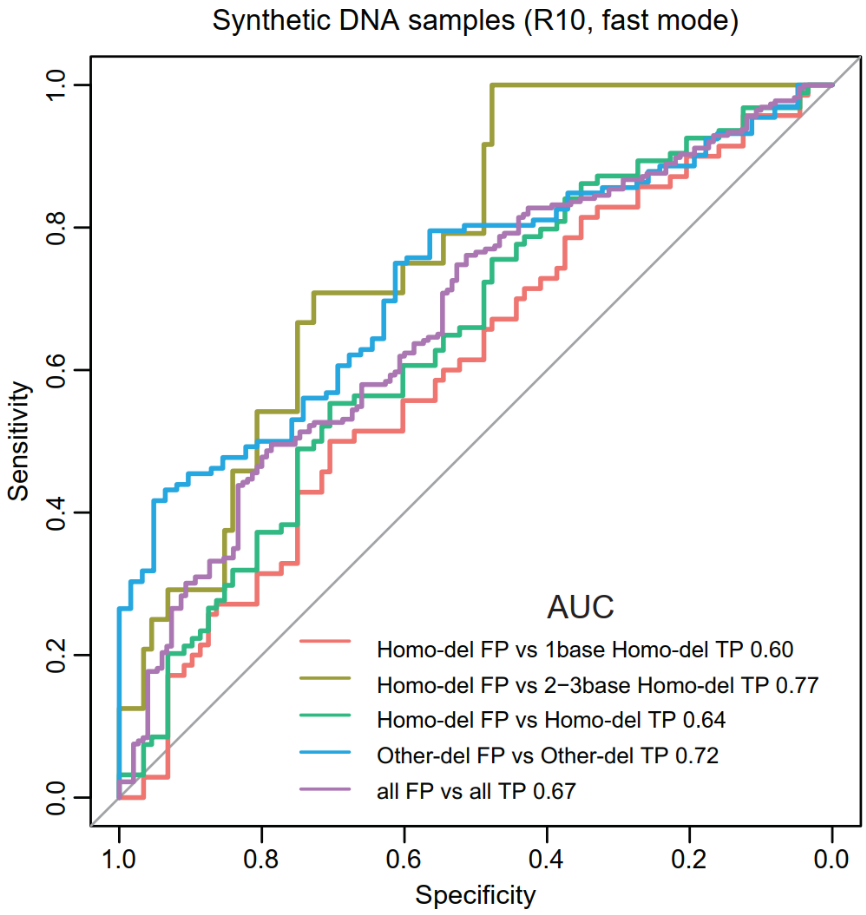

Supplementary Figure S10. The ROCs and AUCs of Q score in distinguishing between FP and TP deletions in R10 direct sequencing data basecalled with the fast model.

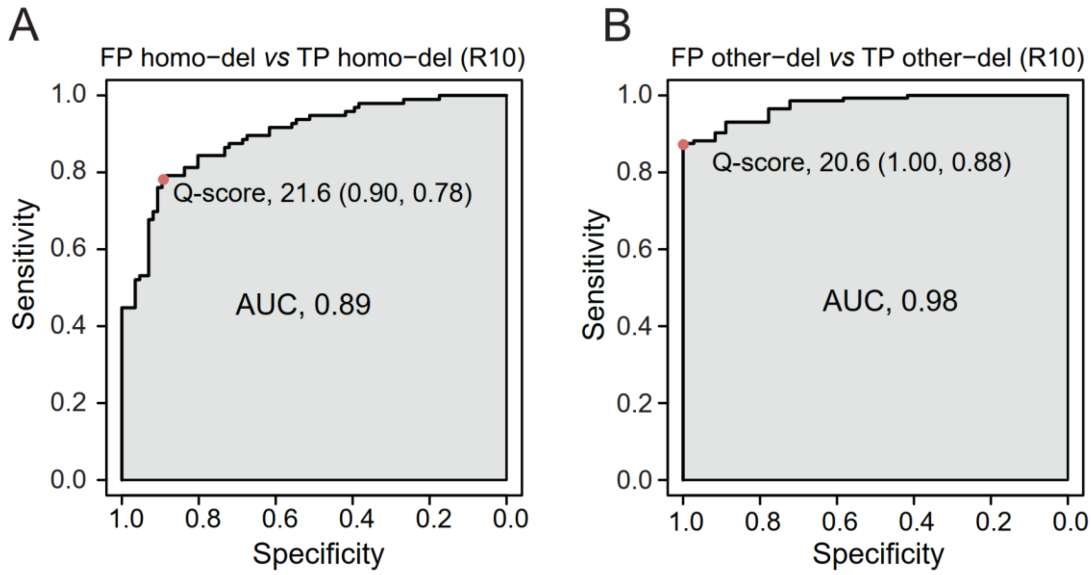

Supplementary Figure S11. Performance assessment of Q score in distinguishing between FP and TP deletions in R10 direct sequencing samples. (A) The ROC of Q score in distinguishing between FP and TP homo-dels. (B) The ROC of Q score in distinguishing between FP and TP other-dels. The best threshold, specificity, sensitivity, and AUC of the HAC model were plotted.

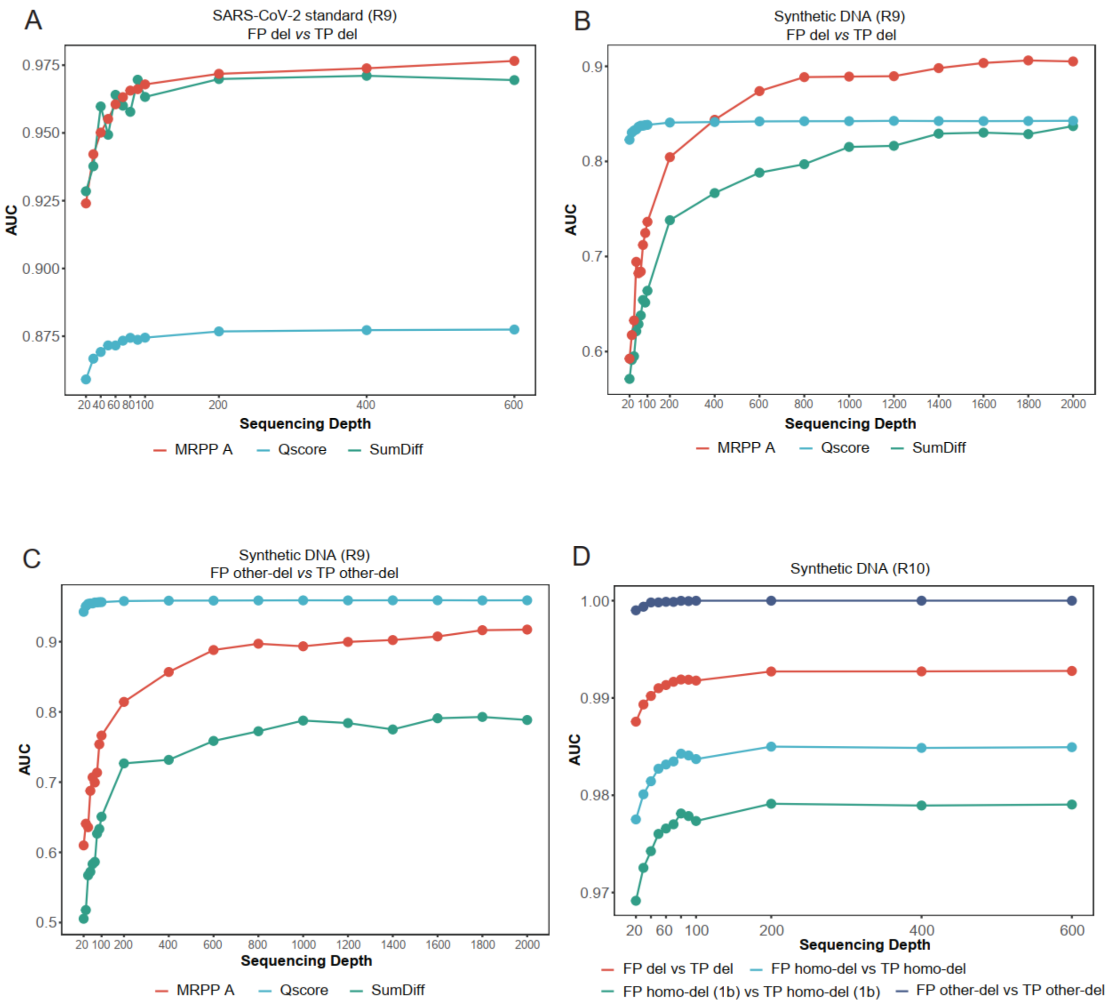

Supplementary Figure S12. The AUCs distinguishing between TP and FP variations across different sequencing depths. (A) The MRPP A- and Q score-derived AUCs corresponding to all deletions at each sequencing depth in WTA sequencing data. (B-C) The MRPP A- and Q score-derived AUCs corresponding to all deletions (B), and other-dels (C) in R9 direct sequencing data. (D) The Q score-derived AUCs corresponding to all deletions, homo-dels, 1-base homo-dels, and other-dels in R10 direct sequencing data. SumDiff: sum of difference.

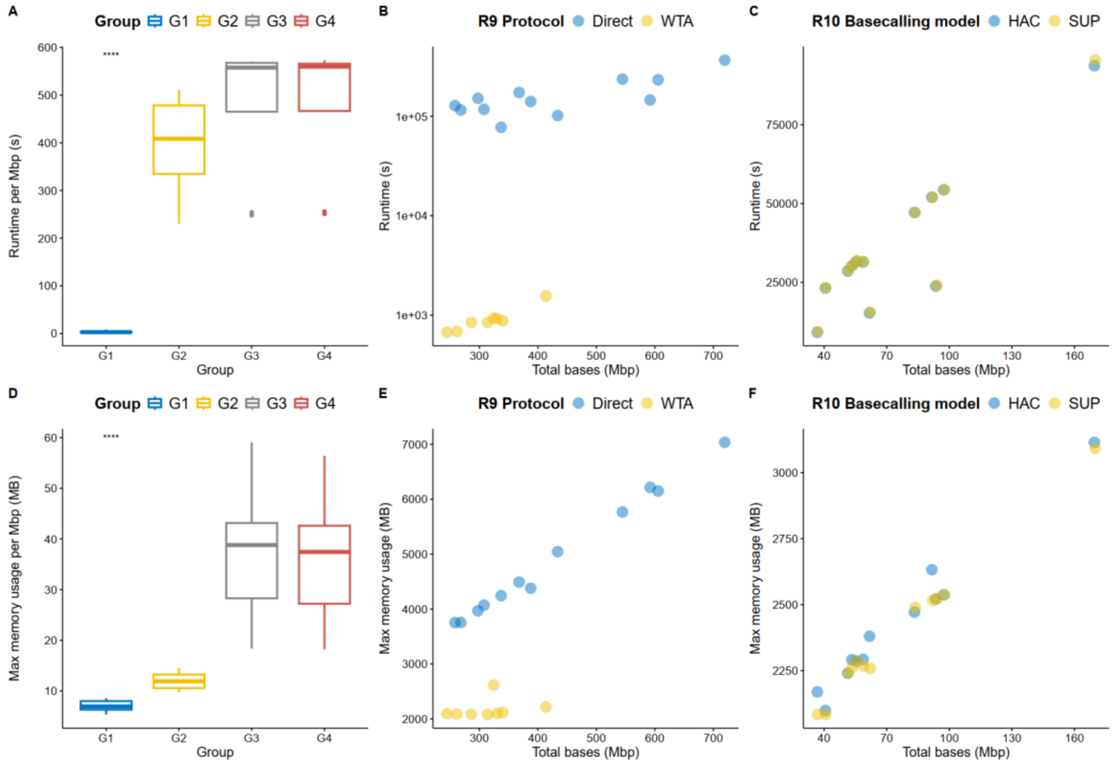

Supplementary Figure S13. The runtime and RAM usage required by LoFreq. (A) The runtime as a function of the wall-clock time (seconds) divided by the number of total basepairs (Runtime per megabasepairs; y-axis) in each group of samples (x-axis). (B) The runtime as a function of the number of total basepairs in R9 sequencing data. WTA: WTA sequencing of SARS-CoV-2 synthetic RNA controls; Direct: direct sequencing of synthetic DNA plasmids. (C) The runtime as a function of the number of total basepairs in R10 sequencing data of synthetic DNA plasmids that are basecalled with the SUP or HAC model. (D) The maximum memory usage as a function of the maximum memory usage (MB) divided by the number of total basepairs (Max memory usage per megabasepairs; y-axis) in each group of samples (x-axis). (E) The maximum memory usage as a function of the number of total basepairs in R9 sequencing data. WTA: WTA sequencing of SARS-CoV-2 synthetic RNA controls; Direct: direct sequencing of synthetic DNA plasmids. (F) The maximum memory usage as a function of the number of total basepairs in R10 sequencing data of synthetic DNA plasmids that are basecalled with the SUP or HAC model. Boxes represent the interquartile range (IQR) between the first and third quartiles (25th and 75th percentiles, respectively). Lines inside denote the median, and whiskers denote the most extreme values within 1.5 times IQR from the first and third quartiles. Outlier values are represented as points. ****P ≤ 0.0001. Each point represents a single sample. G1: SARS-CoV-2 synthetic RNA controls sequenced with R9 flow cell, WTA sequencing protocol, and basecalled with the SUP model (R9+WTA seqeuncing+SUP); G2: synthetic DNA plasmids sequenced with R9 flow cell, direct sequencing protocol, and basecalled with the SUP model (R9+Direct sequencing+SUP); G3: synthetic

DNA plasmids sequenced with R10 flow cell, direct sequencing protocol, and basecalled with the HAC model (R10+Direct sequencing+HAC); G4: synthetic DNA plasmids sequenced with R10 flow cell, direct sequencing protocol, and basecalled with the SUP model (R10+Direct sequencing+SUP).

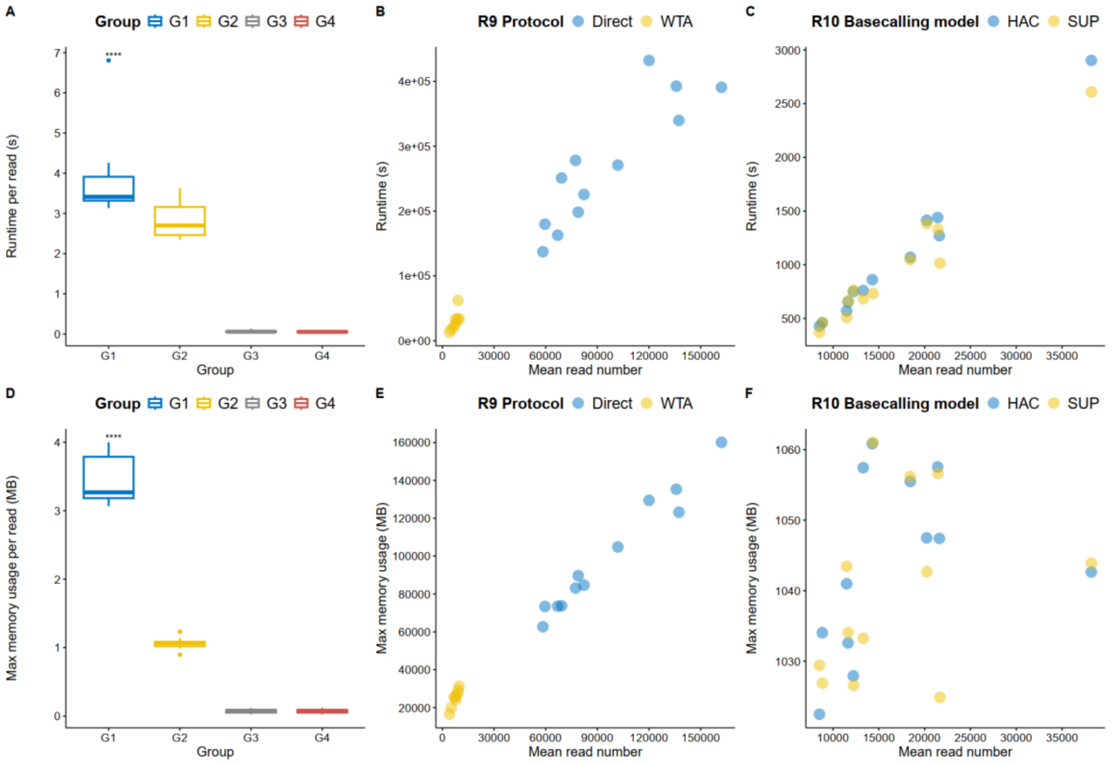

Supplementary Figure S14. The runtime and RAM usage required by Delter. (A) The runtime as a function of the wall-clock time (seconds) divided by the mean number of reads aligned to each deletion variation (Runtime per read; y-axis) in each group of samples (x-axis). (B) The runtime as a function of the mean number of reads aligned to each deletion variation in R9 sequencing data. WTA: WTA sequencing of SARS-CoV-2 synthetic RNA controls; Direct: direct sequencing of synthetic DNA plasmids. (C) The runtime as a function of the mean number of reads aligned to each deletion variation in R10 sequencing data of synthetic DNA plasmids that are basecalled with the SUP or HAC model. (D) The maximum memory usage as a function of the maximum memory usage (MB) divided by the mean number of reads aligned to each deletion variation (Max memory usage per read; y-axis) in each group of samples (x-axis). (E) The maximum memory usage as a function of the mean number of reads aligned to each deletion variation in R9 sequencing data. WTA: WTA sequencing of SARS-CoV-2 synthetic RNA controls; Direct: direct sequencing of synthetic DNA plasmids. (F) The maximum memory usage as a function of the mean number of reads aligned to each deletion variation in R10 sequencing data of synthetic DNA plasmids that are basecalled with the SUP or HAC model. Boxes represent the interquartile range (IQR) between the first and third quartiles (25th and 75th percentiles, respectively). Lines inside denote the median, and whiskers denote the most extreme values within 1.5 times IQR from the first and third quartiles. Outlier values are represented as points. ****P ≤ 0.0001. Each point represents a single sample. G1: SARS-CoV-2 synthetic RNA controls sequenced with R9 flow cell, WTA sequencing protocol, and basecalled with the SUP model (R9+WTA seqeuncing+SUP); G2: synthetic

DNA plasmids sequenced with R9 flow cell, direct sequencing protocol, and basecalled with the SUP model (R9+Direct sequencing+SUP); G3: synthetic DNA plasmids sequenced with R10 flow cell, direct sequencing protocol, and basecalled with the HAC model (R10+Direct sequencing+HAC); G4: synthetic DNA plasmids sequenced with R10 flow cell, direct sequencing protocol, and basecalled with the SUP model (R10+Direct sequencing+SUP).

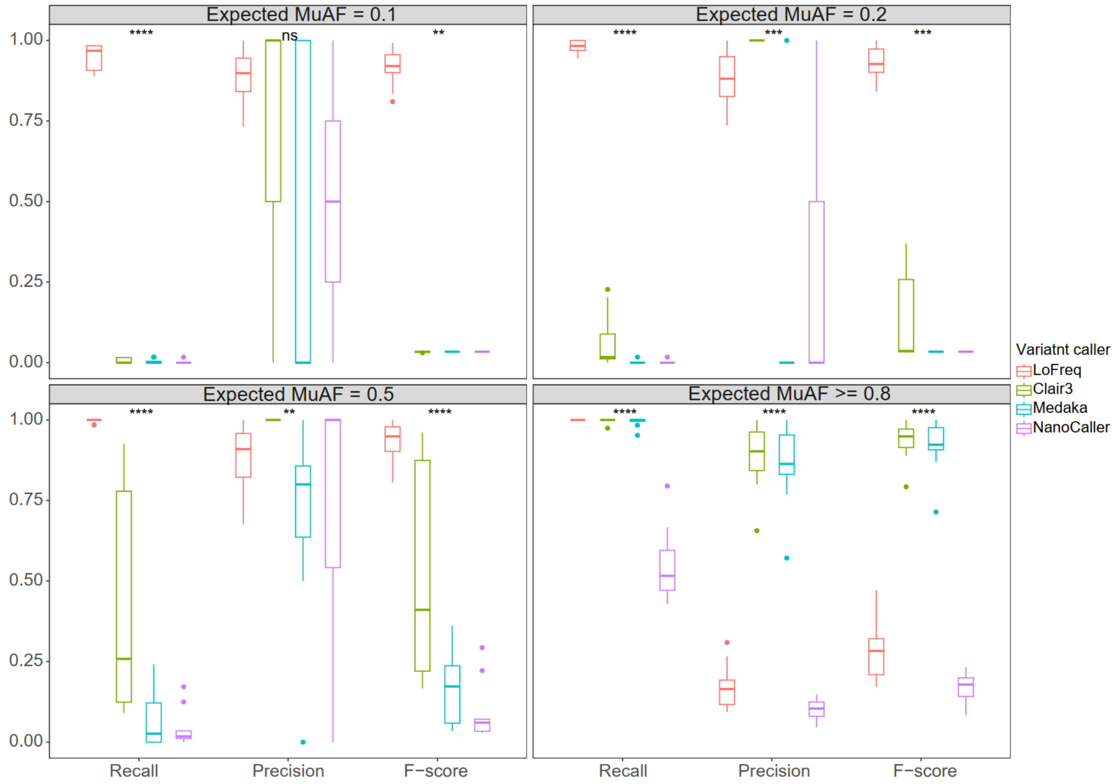

Supplementary Figure S15. Comparison of four variant callers in nanopore sequencing data across different mutated allele frequencies. ns: P > 0.05; **: P ≤ 0.01; ***: P ≤ 0.001; ****: P ≤ 0.0001.
