# Supplementary material for: Characteristics and filtering of low-frequency artificial short deletion variations based on nanopore sequencing
Source: Gigascience. 2025 Mar 21;14:giaf018. doi: 10.1093/gigascience/giaf018 (PMC11927395; doi:10.1093/gigascience/giaf018)
Supplement: giaf018_GIGA-D-24-00312_Revision_2 [file giaf018_giga-d-24-00312_revision_2.pdf]

GigaScience

Characteristics and filtering of low-frequency artificial short deletion variations based on nanopore sequencing

--Manuscript Draft--

**Manuscript Number:**	GIGA-D-24-00312R2
**Full Title:**	Characteristics and filtering of low-frequency artificial short deletion variations based on nanopore sequencing
**Article Type:**	Technical Note
**Funding Information:**	
**Abstract:**	Background<br>Nanopore sequencing is characterized by high portability and long reads, albeit accompanied by systematic errors causing short deletions. Few tools can filter low-frequency artificial deletions, especially in single samples.<br>Results<br>To solve this problem, we first synthesized or purchased 17 DNA/RNA standards for nanopore sequencing with R9 and R10 flowcells to obtain benchmarking datasets. False positive (FP) deletions were prevalent (75.86%-96.26%), while the majority (62.07%-79.68%) were located in homopolymeric regions. The 10-mer base-quality scores (Q scores) and sequencing speeds flanking the FP homopolymeric deletions marginally differed from the true positive (TP) deletions. We thus investigated the raw current signals after normalizing them by length. We found more significant differences in current signals between the reads with and without FP deletions. Indexes including the MRPP A (Multiple Response Permutation Procedure, statistic A), the accumulative difference of normalized current signals, and the Q score were tested for the power of distinguishing between FP and TP deletions. MRPP A outperformed the other indexes in homopolymeric regions and achieved the highest accuracy of 76.73% for challenging 1-base homopolymeric deletions. When sequencing depth was low, the Q score performed better than MRPP A. We developed Delter (Deletion filter) to filter low-frequency FP deletions of nanopore sequencing in single samples, which removed 60.98%-100% artificial homopolymeric deletions in real samples.<br>Conclusions<br>Low-frequency artificial short deletion variations, especially the most challenging homopolymeric deletions, could be effectively filtered by Delter using normalized current signals or Q scores according to the employed sequencing strategies.
**Corresponding Author:**	Fuqiang Ye<br>Huadong Research Institute for Medicine and Biotechniques<br>Nanjing, CHINA
**Corresponding Author Secondary Information:**	
**Corresponding Author's Institution:**	Huadong Research Institute for Medicine and Biotechniques
**Corresponding Author's Secondary Institution:**	
**First Author:**	Fuqiang Ye
**First Author Secondary Information:**	
**Order of Authors:**	Fuqiang Ye<br>Juanjuan Zhu<br>Xiaomin Zhang<br>Jiarong Zhang<br>Zihan Xie<br>Tingting Yang<br>Yifang Han

Powered by Editorial Manager® and ProduXion Manager® from Aries Systems Corporation

	Xiaohong Yang
	Zilin Ren
	Ming Ni
**Order of Authors Secondary Information:**	
**Response to Reviewers:**	Responses to the Editor and Reviewers<br>Editor comments:<br>Response:<br>We have carried out some essential formatting revisions suggested by the Editors.<br><br>Reviewer comments:<br>Reviewer #1:<br>I appreciate the responses to all of my comments and am happy with everything now.<br>Response:<br>Thank you very much for your valuable comments which have significantly improved the manuscript.<br><br>Reviewer #2:<br>Overall the manuscript has been strengthened with additional benchmarks, and the contribution made more comprehensive with the reporting of computational requirements of the tools involved. Reviewer feedback has been addressed diligently and with appropriate care for reporoducibility.<br><br>A minor comment for lines 704-710: "has recall" (plural not necessary); "as it balanced recall and precision" (plural not necessary); "which indicates Clair3 can benefit" (proves is a strong guarantee that would warrant more extensive exploration of Clair3 + Delter).<br>Response:<br>Thank you very much for your valuable comments which have significantly improved the manuscript. We have replaced “has recalls” with “has recall”, and replaced “has precisions” with “has precision”. In addition, “as it balanced recalls and precisions” has been replaced with “as it balanced recall and precision”. The statement “which proves Clair3 could benefit” has also been modified to “which indicates Clair3 can benefit”.
**Additional Information:**	
**Question**	**Response**
Are you submitting this manuscript to a special series or article collection?	No
**Experimental design and statistics**<br><br>Full details of the experimental design and statistical methods used should be given in the Methods section, as detailed in our Minimum Standards Reporting Checklist. Information essential to interpreting the data presented should be made available in the figure legends.<br><br>Have you included all the information requested in your manuscript?	Yes
**Resources**	Yes

A description of all resources used, including antibodies, cell lines, animals and software tools, with enough information to allow them to be uniquely identified, should be included in the Methods section. Authors are strongly encouraged to cite Research Resource Identifiers (RRIDs) for antibodies, model organisms and tools, where possible.<br><br>Have you included the information requested as detailed in our Minimum Standards Reporting Checklist?	
**Availability of data and materials**<br><br>All datasets and code on which the conclusions of the paper rely must be either included in your submission or deposited in publicly available repositories (where available and ethically appropriate), referencing such data using a unique identifier in the references and in the "Availability of Data and Materials" section of your manuscript.<br><br>Have you have met the above requirement as detailed in our Minimum Standards Reporting Checklist?	Yes

Powered by Editorial Manager® and ProduXion Manager® from Aries Systems Corporation

Manuscript

Click here to access/download;Manuscript;homo-20241217-Gigascience R2 clean 250210.docx

Characteristics and filtering of low-frequency artificial short deletion variations based on nanopore sequencing

Fuqiang Ye[1,†,‡], Juanjuan Zhu[2,†,‡], Xiaomin Zhang[3,†,‡], Jiarong Zhang[3,4], Zihan Xie[3,5], Tingting Yang[3,4], Yifang Han[1], Xiaohong Yang[1], Zilin Ren[6,7,*], Ming Ni[3,*]

[1]Huadong Research Institute for Medicine and Biotechniques, Nanjing 210002, People's Republic of China

[2]School of Life Science and Technology, China Pharmaceutical University, Nanjing 211198, People's Republic of China

[3]Department of Advanced & Interdisciplinary Biotechnology, Academy of Military Medical Sciences, Beijing 100850, People's Republic of China

[4]School of Forensic Medicine, Shanxi Medical University, Jinzhong 030600, People's Republic of China

[5]College of Life Science and Technology, Beijing University of Chemical Technology, Beijing 100029, People's Republic of China

[6]Changchun Veterinary Research Institute, Chinese Academy of Agricultural Sciences, State Key Laboratory of Pathogen and Biosecurity, Key Laboratory of Jilin Province for Zoonosis Prevention and Control, Changchun 130122, People's Republic of China

[7]School of Information Science and Technology, Northeast Normal University, Changchun 130117, People's Republic of China

† Co-first authors

‡Contributed equally to this work

* To whom correspondence should be addressed. Email: niming@bmi.ac.cn. Correspondence may be also addressed to Zi-lin Ren. Email: zilin.ren@outlook.com.

Abstract

Background

Nanopore sequencing is characterized by high portability and long reads, albeit accompanied by systematic errors causing short deletions. Few tools can filter low-frequency artificial deletions, especially in single samples.

Results

To solve this problem, we first synthesized or purchased 17 DNA/RNA standards for nanopore sequencing with R9 and R10 flowcells to obtain benchmarking datasets. False positive (FP) deletions were prevalent (75.86%-96.26%), while the majority (62.07%-79.68%) were located in homopolymeric regions. The 10-mer base-quality scores (Q scores) and sequencing speeds flanking the FP homopolymeric deletions marginally differed from the true positive (TP) deletions. We thus investigated the raw current signals after normalizing them by length. We found more significant differences in current signals between the reads with and without FP deletions. Indexes including the MRPP A (Multiple Response Permutation Procedure, statistic A), the accumulative difference of normalized current signals, and the Q score were tested for the power of distinguishing between FP and TP deletions. MRPP A outperformed the other indexes in homopolymeric regions and achieved the highest accuracy of 76.73% for challenging 1-base homopolymeric deletions. When sequencing depth was low, the Q score performed better than MRPP A. We developed Delter (Deletion filter) to filter low-frequency FP deletions of nanopore sequencing in single samples, which removed 60.98%-100% artificial homopolymeric deletions in real samples.

Conclusions

Low-frequency artificial short deletion variations, especially the most challenging homopolymeric deletions, could be effectively filtered by Delter using normalized current signals or Q scores according to the employed sequencing strategies.

Keywords: Nanopore sequencing, low-frequency deletions, filtering

Running title：Characteristics and filtering of artificial deletions of nanopore sequencing

Introduction

Nanopore sequencing is distinguished by its high portability, compared to other commercially available sequencing technologies such as the single molecule real-time (SMRT) sequencing by PacBio (CA, USA) and massive parallel sequencing (MPS) by Illumina (CA, USA) and MGI Tech (Shenzhen, China). The smallest sequencer now is the MinION Mk1B (Oxford Nanopore Technologies, Oxford, UK), which weighs only 87 grams. It can yield Gb-level sequencing data in a single run and has low requirements for environmental conditions. These features make MinION Mk1B well-suited for in-field sequencing applications such as viral genomic surveillance during epidemics and biodiversity surveillance [1-3]. On the other hand, the lengths of nanopore sequencing reads are primarily determined by the DNA or RNA molecules passing through nanopores. Nanopore sequencing is widely used for genome and transcriptome assembling [4-11] and long haplotype phasing [12, 13].

Despite the benefits in portability and sequencing length, nanopore sequencing still exhibits relatively higher noise than MPS and SMRT sequencing [14, 15]. Compared to the initial version of nanopore sequencing devices, the accuracy of current sequencers has been notably improved by engineered pore proteins [14, 16] and deep-learning-based basecalling tools [14, 17]. Multiple studies have demonstrated that using nanopore sequencing enables the acquisition of reliable consensus genomes and variations for viruses, bacteria, and human [8, 15, 18-21]. However, when heterogeneity of genetic materials exists, such as in viral quasi-species, heterogenous bacterial colonies, and tumors with heterogeneity, nanopore sequencing still needs improvement in identifying the low-frequency variations [14, 19, 22].

Moreover, nanopore sequencing is more prone to errors in short insertions and deletions (indels), especially in low-complexity regions like homopolymers, compared to single nucleotide variations (SNVs) [23-28]. Stancu *et al.* reported a 2.6-fold increase in deletion errors for sequences overlapping with homopolymers [25]. Delahaye *et al.* found that nearly 50% of nanopore sequencing errors were attributed to homopolymers [27]. A recent study benchmarked seven nanopore sequencing base-caller models and observed median homopolymer error rates of 14.9%-44.5% [28]. The high error rates in homopolymeric regions can impose limitations on the application of nanopore sequencing. For instance, artificial deletion variations with low frequencies (< 0.5) in cancer-related genomic tests are more prone to be misclassified as pathogenic or likely pathogenic than artificial SNVs. Accurately detecting low-frequency variations is also pivotal in identifying intra-host heterogeneity of pathogens, which is crucial for studying the micro-evolution, adaption, and recombination of viruses or bacteria [29-31]. Nonetheless, several studies have suggested that nanopore sequencing is unsuitable for detecting intra-host indels and SNVs due to the high levels of low-frequency errors [18, 19].

To date, there is a lack of methods to filter artificial low-frequency variations for nanopore sequencing. Recently, Liu *et al.* reported a tool named Variabel that employs longitudinal or cross-sectional samples to recover low-frequency intra-host variations [26]. Variabel can identify low-frequency variations with a < 0.5 allele frequency, but its performance in differentiating genuine and artificial indels in homopolymeric regions was not assessed. To our knowledge, no

method or tool has been proposed for filtering artificial low-frequency indels applicable to single-sample nanopore sequencing. The errors in nanopore sequencing, particularly those in homopolymeric regions, are primarily ascribed to the basecalling process, in which raw electric current singles are converted into nucleotide sequences [28]. It has been reported that a fine-tuned or specially trained model for a selected set of nucleotide sequences could potentially reduce the false positive rates of basecaller [32]. However, there has been no comprehensive investigation of low-frequency errors in nanopore sequencing.

In this study, we employed the R9 and R10 flow cells and chemistries of nanopore sequencing to sequence synthetic nucleotides. Our results show that > 96.00% of the artificial variations had a frequency < 0.3, and most (73.00%) were short deletions in homopolymeric regions. We compared the raw current signals, base-quality scores (Q scores), and passing-pore sequencing speeds of the reads with and without deletions, and characterized their differences. We developed a tool named Delter (Deletion filter) to distinguish between false and true low-frequency short deletions identified using nanopore sequencing (Figure 1).

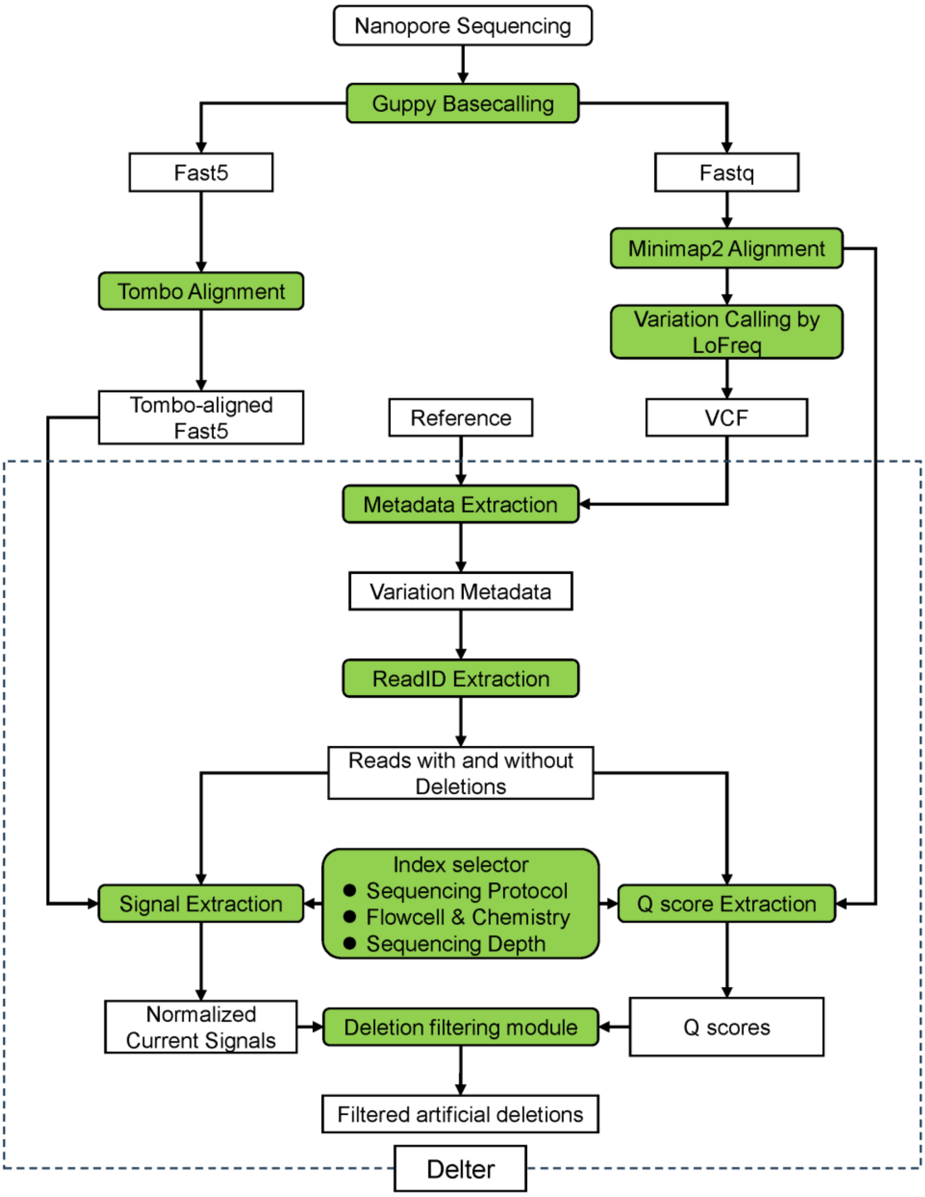

Figure 1. Workflow of Delter for filtering FP deletions in R9 and R10 nanopore sequencing. Delter is organized as a Snakemake workflow. It is composed of 6 functional modules: (1) Metadata extraction module; (2) ReadID extraction module; (3) Index selector; (4) Signal extraction module; (5) Q score extraction module; (6) Deletion filtering module. Details are listed in the section of "Materials and Methods".

Materials and Methods

Synthetic DNA and RNA sequences

Nine SARS-CoV-2 synthetic RNA genome controls (Twist Bioscience, USA), including the wild-type Wuhan-Hu-1 strain (GenBank accession MN908947.3) and variants from Alpha, Beta, Delta, Epsilon, Iota, Kappa, Omicron BA.1, and Omicron BA.2 lineages were purchased (Supplementary Table S1). With the genome of the Wuhan-Hu-1 strain as a reference, other SARS-CoV-2 variants contain 306 deletion variations with lengths ranging from 3 to 9 nucleotides (nts, Supplementary Table S2).

In addition, wild-type and mutated nucleotide sequences of the S gene of SARS-CoV-2 (GenBank accession MN908947.3), Penton gene of Human adenovirus subtype 55 (HAdV55, GenBank accession MK886831.1), 16S rDNA gene of *Escherichia coli* (Strain: Castellani and Chalmers 1919, 01485cm, NRRL accession B-1109) and an assembly contig of *Saccharomyces cerevisiae* (Strain: Saccharomyces cerevisiae Meyen ex E. C. Hansen (1883) ATCC 9763, NRRL accession Y-567) were synthesized in plasmids by Sangon Biotech Co., Ltd. (Shanghai, China) (Supplementary Table S3). The mutated sequences were designed to contain 242 deletions of 1 to 3 nts, which were evenly distributed in homopolymeric and non-homopolymeric regions (Supplementary Table S4).

Nanopore Sequencing and Basecalling

SARS-CoV-2 synthetic RNA controls were reversely transcribed with Qiagen Whole Transcriptome Amplification Kit (Cat no. 207043, Qiagen, Germany) following the manufacturer's instruction. The cDNA products and synthetic DNA plasmids were employed as inputs to prepare libraries with Rapid Barcoding Kit (SQK-RBK004, Oxford Nanopore Technologies, UK) and sequenced by using a MinION Mk1B sequencer with the R9.4.1 (FLO-MIN106, Oxford Nanopore Technologies, UK) and R10.4.1 flow cells (FLO-MIN114, Oxford Nanopore Technologies, UK) according to the manufacturer's protocols.

The MinKNOW software (v22.03.5, v22.08.9, or v22.10.10, Oxford Nanopore Technologies) was employed to the sequencing run, and Guppy (v6.0.6, v6.2.11, or v6.3.9, Oxford Nanopore Technologies), an integrated component of MinKNOW, was used for basecalling throughout sequencing runs. For sequencing with R9.4.1 flow cells, re-basecalling was conducted using Guppy (v6.2.1, RRID:SCR_023196) with the super accuracy (SUP) model. For the R10 sequencing run, the fast, high accuracy (HAC), and SUP basecalling models were utilized.

Quality Control and Alignment of base-called reads

The sequencing adapters were trimmed by using Porechop (v0.2.4, RRID:SCR_016967). NanoFilt (v2.8.0, RRID:SCR_016966) [33] was then used to filter reads with undesirable lengths and low quality scores (-q 8 --length 100) and to trim ten bases from 5'/3' ends of the reads. Minimap2 (v2.24, RRID:SCR_018550) [34] was used for alignment of clean reads to reference sequences, with the preset parameters for nanopore sequencing data (-ax map-ont). Samtools (v1.13, RRID:SCR_005227) [35] was employed for downstream analyses of the alignments. The unmatched fragments marked as soft-clipped in BAM file of aligned reads were trimmed using in-house scripts.

Variation Calling and Filtering

Nanopore sequencing variations were identified using LoFreq2 (v2.1.5, RRID:SCR_013054), which is applicable for analyzing nanopore sequencing data to detect low-frequency variations [36]. First, with the "lofreq indelqual" parameter, indel quality scores were added to the BAM files. Then, the "lofreq call-parallel" command was used to call variations with the following parameters "–no-default-filter –call-indels". The candidate variations were filtered using the

“lofreq filter” command with parameters of "lofreq filter --cov-min 100 --af-min 0.05 --sb-alpha 0.01 --sb-incl-indels". For sequencing data of synthetic SARS-CoV-2 controls that underwent whole transcriptome amplification, variations were also identified using VarScan2 (v2.4.4, RRID:SCR_006849) [37] and Medaka (v1.7.3) [38] with default parameters. Clair3 (v1.0.10, RRID:SCR_026063) [39] and NanoCaller (v3.6.0) [40] were employed for comparison of different variant callers.

Aligning Electric Current Signals to Ground Truth Nucleotide Sequences

The raw R9 sequencing data containing the electric current signal level data (current signals or squiggles) and the associated base-calls were stored in ‘fast5’ formatted files. The ‘multi_to_single_fast5’ command from the ont-fast5-api python package (v4.1.1) [41] was performed to spilt multiple-read fast5 files into single-read fast5 files. Then, Tombo (v1.5.1, RRID:SCR_024388) was utilized to load single-read fast5 file and assign current measurements in the squiggle to each base of the read via alignment to the reference sequence with the ‘resquiggle’ command.

The squiggles of 10 bases or 20 bases flanking each variation were extracted using in-house scripts to compare between reads with and without artificial deletions. As the sampling rate of the MinION sequencer is 4000 times per second, the time interval between two consecutive current measurements in the squiggle is fixed. The real-time sequencing or translocation speed of the DNA molecules passing through the nanopores (number of current measurements per base) was determined by dividing the number of current measurements (signal lengths) of the relevant read fragments by the number of bases.

The passing-pore sequencing speeds of DNA molecules are highly diverse. Namely, the same number of nucleotides can produce different lengths of current signals. Therefore, before further analysis, these current signals were normalized by length using a binning approach. The mean values of the current measurements assigned to the same bin were utilized. The sums of difference values (accumulative differences) between the normalized signals of reads with and without deletions were also calculated.

Subsampling approach to determine thresholds

Our method was evaluated under different sequencing depths using a subsampling approach. For each variation, we randomly chose N (range: 20 to 2000) forward and reverse-aligned reads supporting the reference and non-reference alleles, respectively. Namely, N forward-aligned reads supporting the reference allele, N reverse-aligned reads supporting the reference allele, N forward-aligned reads supporting the non-reference allele, and N reverse-aligned reads supporting the non-reference allele (strand-specific sequencing depth). Thus, 80 to 8000 aligned reads per variation were subsampled when available. The AUCs, sensitivities, and specificities corresponding to each sequencing depth were calculated. The threshold with the highest sum of sensitivity and specificity was used as the default threshold for filtering.

Statistical Analyses and Visualization

The R project (v4.2.2) [42] was employed for the statistical analyses and visualization. To compare the normalized signals with equal lengths from reads with and without variations, we used three inter-group difference analysis methods, including analysis of similarities (ANOSIM), multi-response permutation procedure (MRPP), and permutational multivariate analysis of variance (ADONIS2). The R package Vegan (v2.6-4, RRID:SCR_011950) was utilized for the ANOSIM, MRPP, and ADONIS2 calculation. The Kruskal-Wallis rank-sum test was used for inter-group comparison. P values were adjusted with the Benjamini and Hochberg method when necessary. The receiver operating characteristic (ROC) curve analysis was conducted to assess the performance of filtering artificial variations using the R package pROC (v1.18.2, RRID:SCR_024286). The R packages ggplot2 (v3.4.1, RRID:SCR_014601), ggpubr (v0.6.0, RRID:SCR_021139), ggsci (v2.9) [43], and ComplexHeatmap (v2.14.0, RRID:SCR_017270) were implemented for visualization.

Implementation of the filter for removing artificial deletions

Delter is organized as a Snakemake workflow. It is composed of 6 functional modules: (1) Metadata extraction module. This module uses a VCF file output by LoFreq and reference sequence as inputs to generate the variation metadata including deletion type (homo-dels or other-dels), deletion length, and the starting and ending positions; (2) ReadID extraction module. Its main function is to get read lists containing deletions and with no deletions; (3) Index selector. This core module automatically selects appropriate index(es) depending on the sequencing protocol, flowcell/chemistry, and sequencing depth; (4) Signal extraction module. If MRPP A is chosen for downstream analyses, this module will extract the raw current signals of N bases flanking each deletion variation, which are preprocessed to normalized current signals using a binning approach; (5) Q score extraction module. If Q score is selected, it will output base qualities of 10-mer read region of deletion variations; (6) Deletion filtering module. This module bundled several functions to calculate MRPP A and average 10-mer Q scores. It also filters and marks artificial deletions in the final output.

The filter takes several files as inputs: (1) the VCF file output by LoFreq; (2) the sorted BAM files storing alignment of nanopore reads to reference sequence; (3) the reference sequence; (4) the directory storing Tombo-resquiggled single-read fast5 files when R9 flow cell and chemistry are employed. Users should also provide the sequencing protocol (amplicon or direct), flowcell/chemistry (R9 or R10), strand-specific sequencing depth for subsampling, the directory storing the final results, and the base number flanking each variation to extract Q scores and current signals.

External validation using real samples

Our method was validated in sequencing data of HAdV and microbial standard samples. We first synthesized plasmids containing full-length Fiber, Penton, and Hexon genes from different HAdV subtypes including HAdV11, 14, and 55 (n=7, Supplementary Table S5). These genes natively contain five real deletions when compared to references. Partial gene fragments (amplicons)

covering true deletions were also amplified using PCR primers (n=9). The samples were mixed and sequenced using R9.4.1 flow cell (n=9). ZymoBIOMICS Gut Microbiome Standard (Cat no. D6331, Zymo Research, USA) containing varying bacterial cell contents was further sequenced with R9.4.1 flow cell and processed. As these bacteria should not have any true variations, all of the variations output by LoFreq are identified as negative. In order to assess the applicability of the tool for metagenomic scenarios, we also evaluated the pipeline in the case of the co-existence of closely related bacterial strains. The microbial standard D6331 contains five strains of *Escherichia coli* (*E. coli*), namely, B-1109, B-766, B-2207, B-3008, and JM109. We randomly selected the strain B-1109 as the reference genome, and then evaluated the similarities between the other four strains and the reference using fastANI (v1.33, RRID:SCR_021091). The average nucleotide identities range from 98.46% to 99.52%. DNAdiff (v1.3) and GSAlign (v1.0.22) [44] were employed to call variations of each strain relative to the reference B-1109 separately. The shared deletion variations reported by both tools were merged to constitute a list of true deletions (n=236). The D6331 nanopore sequencing data was then aligned to the B-1109 reference genome. The VCF was called with LoFreq and used as input for the Delter workflow.

Moreover, three public datasets available on the NCBI SRA database were included for further assessment. The African swine fever virus (ASFV) dataset has one Nanopore R10 run and four paired Illumina MiSeq runs (NCBI accession number: PRJNA1096272). The *Pseudomonas aeruginosa* (*P. aeruginosa*) PAO1 dataset contains R10 SUP data generated by GridION (SRA accession number: ERR8958864) and paired MiSeq data (ERR9285397). As the GridION dataset has a large number of reads (n > 630,000), only the first 120,000 reads were selected for downstream analyses. The *Brucella suis* (*B. suis*) dataset has paired Nanopore R9 (ERR10828735) and Illumina MiSeq (ERR10820713) sequencing data. After quality control with NanoFilt (nanopore data) or fastp (MiSeq data, v0.22.0, RRID:SCR_016962), all the sequencing data were aligned to the reference genome of ASFV (NC_044945.1), *P. aeruginosa* PAO1 (NC_002516.2) or *B. suis* (NC_004310.3) with Minimap2 or bwa (v0.7.17, RRID:SCR_010910). For Illumina datasets, variations were called using LoFreq with a minimum depth of 20 and a minimum allele frequency of 0.05. Variation calls on Illumina sequencing runs were used as real variations. For nanopore datasets, variations were called using the same parameters as mentioned above.

Results

Nanopore sequencing of synthetic samples

We obtained a total of 17 chemically synthesized RNA and DNA samples for nanopore sequencing, covering both wildtypes and the corresponding mutants (Supplementary Table S1-S4). Nine synthetic RNA samples (~30kb) were SARS-CoV-2 standards, among which eight are variants of interest or concern. Due to their low copy numbers (5000 copies per standard), they underwent whole transcriptome amplification (WTA) before nanopore sequencing (referred to as WTA sequencing). Eight synthetic DNA samples contained the sequences from SARS-CoV-2, Human adenovirus subtype 55 (HAdV55), *Escherichia coli*,

and *Saccharomyces cerevisiae* (length ranged from 1468 to 1674), and the mutants carried designed deletion variations. The mutants and their corresponding wildtypes were respectively mixed with ratios of 1:9, 1:4, and 1:1 to mimic low-frequency variations (Supplementary Figure S1). The synthetic DNA samples from plasmids were directly sequenced without amplification (referred to as direct sequencing). Three independent sequencing runs containing 33 synthetic samples were conducted using the MinION sequencer Mk1B with R9 and R10 flow cells, and a total of 9.3 Gb clean sequencing data were yielded after quality control (Supplementary Table S6). WTA sequencing samples generated shorter reads (N50: 1137-1656 bases) than direct sequencing ones (N50: 4131-4349 bases).

The majority of artificial variations were deletions

Small variations with a ≥ 0.05 mutated allele frequency (MuAF) were identified for data generated by WTA sequencing and direct sequencing with R9 and R10 flow cells. The recall rates of true positive (TP) variations were high, which were 99.67% (305 out of 306) for the WTA sequencing and 97.80% (R9, 710 of 726)-98.07% (R10, 712 out of 726) for the directing sequencing. Most false negatives were attributed to low sequencing depth or marginal MuAFs near the 0.05 threshold.

Abundant artificial (false positive, FP) low-frequency variations were identified (Figure 2A-C). Among all the FP types, the small deletions located in ≥ 3-base homopolymeric regions (denoted as homo-dels) were remarkably dominating (62.07%-79.68%), followed by the deletions in the non-homopolymeric regions (other-dels, 10.40%-16.58%). FP insertions and SNVs took a relatively small proportion of all the FPs. Most FP SNVs of WTA sequencing could be filtered by trimming of read ends [18, 19]. We trimmed 10 bases from both ends of the aligned fragment of reads and reduced 77.11% FP SNVs of WTA sequencing (Figure 2A). Other-dels and insertions were also reduced (22.93% and 14.89%, respectively); whereas only 0.59% FP homo-dels were excluded by the trimming (Figure 2A). In contrast, the trimming led to higher FP ratios in direct sequencing samples (Figure 2B-C), which might be due to the increase of marginal MuAFs to > 0.05. This result was consistent using different variant callers (Supplementary Figure S2). Different basecalling models (fast, high accuracy HAC, and super accuracy SUP) led to diverse FP ratios in R10 direct sequencing samples (Figure 2C; Supplementary Figure S3). The samples sequenced with R10 flow cell generated fewer FP variations than those with R9 flow cell (SUP: 24/172=13.95%; HAC: 85/172=49.42%).

We also investigated how the FP ratios varied as the MuAF thresholds grew. As shown in Figure 2D, different sequencing strategies had similar trends. The majority (> 96.00%) of FP variations had a low MuAF < 0.3. FP variations were usually shared in the datasets of highly homologous samples with identical sequencing strategies. In the WTA sequencing dataset of the SARS-CoV-2 standards, a total of 233 genomic loci were found to have FP variations, of which 25.32% and 47.21% were identified in all or at least 50% of samples (Figure 2E). For the direct sequencing samples, the same FP variations were also identified in mixtures derived from different mutant/wildtype ratios (Figure 2F-G). There were 14 homo-dels shared by both R9 and R10 direct sequencing

samples, while none of other-dels or insertions were shared, indicating the inherent systematic errors in nanopore sequencing despite the flow cell and chemistry.

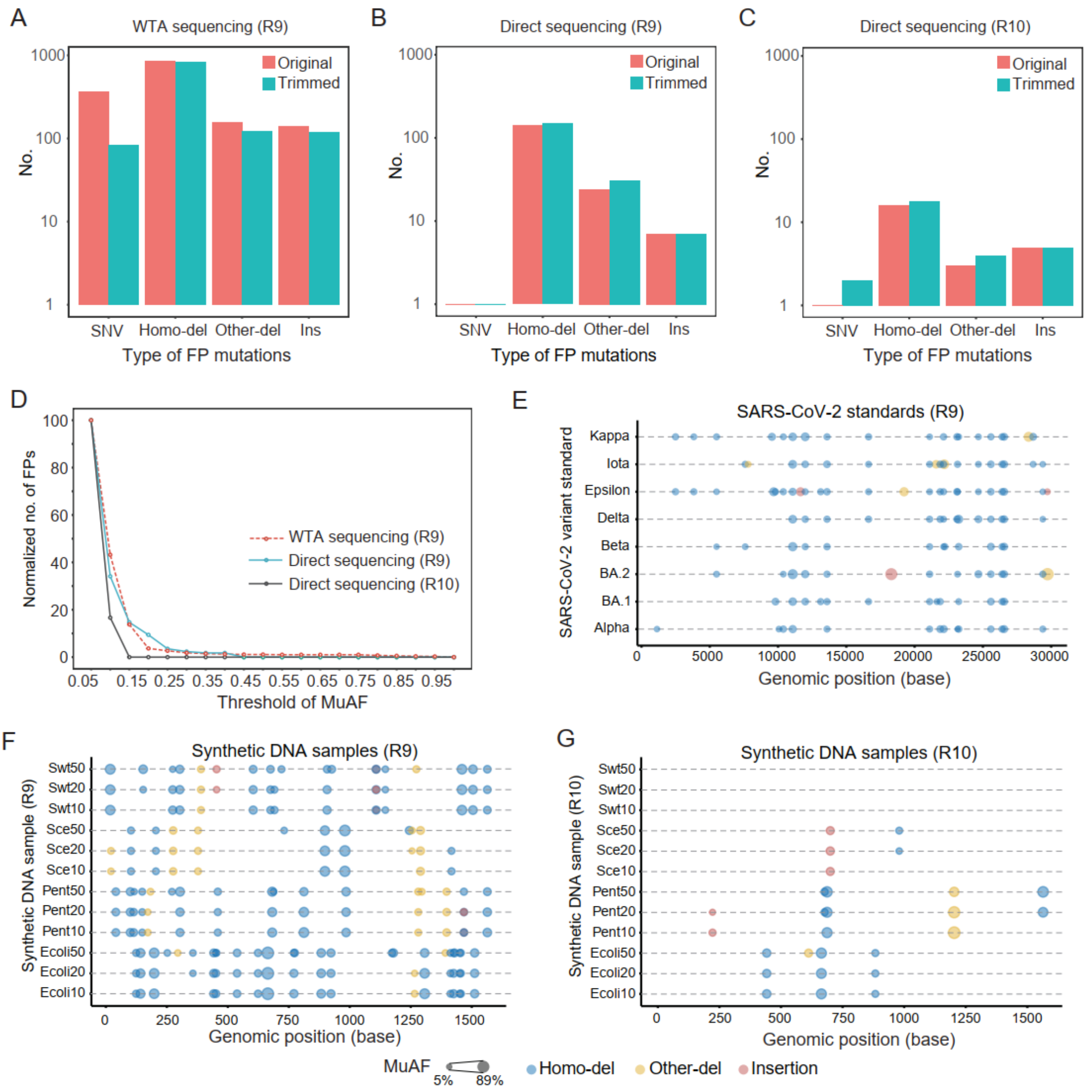

Figure 2. False positive variations in different sequencing strategies with ONT R9 and R10 flow cells and chemistries. (A-C) The bar plots of four types of artificial variations before (red) and after (blue) trimming bases in ONT R9 WTA sequencing data (A), R9 direct sequencing data (B), and R10 direct sequencing data basecalled with the SUP model (C). The y-axis was log10 transfromed. (D) The normalized FP counts at each MuAF threshold relative to those at MuAF=0.05. (E-G) The genomic distributions of FP variations in R9 WTA sequencing data (E), R9 direct sequencing data (F), and R10 direct sequencing data basecalled with the SUP model (G), only deletions and insertions were plotted. FP variations with MuAF ≥ 0.15 in SARS-CoV-2 variants were selected to display for better visualization. Swt: wildtype SARS-CoV-2; Ecoli: *Escherichia coli*; Pent: Human adenovirus subtype 55; Sce: *Saccharomyces cerevisiae*.

A biased Q score and sequencing speed distribution of FP deletions compared to TP deletions

Deletion variations comprise the highest proportion, while SNVs and insertions were relatively low, so we focused mainly on homo-dels and other-dels. The 10-mer average Q scores (upstream and downstream of five bases) flanking each variation were calculated to compare between reads with and without deletions. Reads containing FP homo-dels and other-dels had lower Q scores than reads with no deletions (Figure 3A-C, Supplementary Figure S4A). In contrast, reads containing TP deletion variations had a nearly identical distribution of Q scores relative to reads without deletions. FP homo-dels from different sequencing strategies had diverse Q score distribution compared to other-dels. Moreover, the differences between reads with and without deletions were negligible in the FP homo-dels derived from R10 direct sequencing samples basecalled with the fast basecalling model (Supplementary Figure S4B). Notably, the differences between reads with and without homo-dels were minor relative to those in other-dels, which indicated the difficulty in distinguishing between FP homo-dels and TP deletions.

High room temperature could lead to abnormal translocation speeds of templates going through nanopore proteins and further generate poorer base qualities. We thus analyzed the sequencing speeds of FP and TP deletions. The electric current signal level data (current signals or squiggles) of 10 or 20 bases (10-mer or 20-mer current signals) flanking each deletion variation were extracted. FP deletion variations were observed to have fewer numbers of current measurements (sampling points) per base, namely higher sequencing speeds, than reads without deletions (Figure 3D-E). In contrast, TP deletion variations differed slightly from reads without deletions. At the scale of whole read, FP and TP deletion variations had no significant differences in sequencing speeds (Supplementary Figure S5).

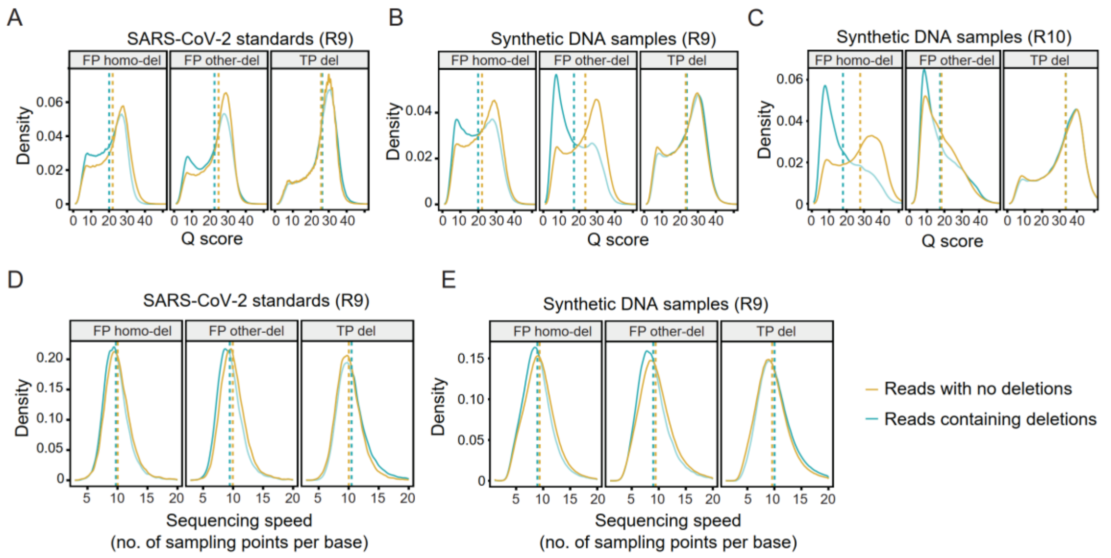

Figure 3. The characteristics of Q score and sequencing speed of FP variations. (A-C) The comparisons of Q scores between reads containing deletion variations and reads with no deletions in R9 WTA sequencing data (A), R9 direct sequencing data (B), and R10 direct sequencing data basecalled with

theSUP model (C). The dashed lines represented the mean values of Q scores. (D-E) The comparisons of sequencing speeds between reads containing deletion variations and reads with no deletions in R9 WTA sequencing data (D) and R9 direct sequencing data (E). The dashed lines represented the mean values of speeds. Sequencing speed equals the division of the number of current measurements by the base number.

Remarkable differences between current signals of R9 FP and TP deletions

Although FP deletions had more significant differences in Q score than sequencing speed, the Q score alone might be insufficient to separate FP homo-dels from true deletions in R9 sequencing data. As Q scores were directly correlated with the basecalling of current signals, we further characterized the raw signals of FP and TP deletions.

The 10-mer current signals flanking FP deletion variations were further processed to inspect whether remarkable differences exist when compared to reads without deletions. As the counts of current measurements (signal lengths) of each variation weren't equal and sequencing speeds had no close relationship with FP or TP deletions, the current signals first underwent binning-based normalization preprocesses to even lengths. The current measurements in each bin were then averaged. The current signals of reads supporting FP deletion variations were found to have slight differences relative to reads without deletions, regardless of homo-dels or other-dels (Figure 4A and Supplementary Figure S6A). However, TP deletions had remarkable discrepancies between reads with and without deletions (Figure 4B and Supplementary Figure S6B). Notably, the differences enlarged as the deletion types varied from homo-dels to other-dels. Moreover, the differences grew bigger when more bases were deleted. The sums of difference values (accumulative differences) between the normalized signals of reads with and without deletions were then calculated. FP deletions were observed to have lower sums than TP deletions (Figure 4C-F), and homo-dels had lower sums than other-dels (Supplementary Figure S7). The differences between TP and FP deletions in current signals were more significant than those observed in Q score and sequencing speed.

The normalized equal signal lengths were also suitable for downstream inter-group difference detection methods. ANOSIM, MRPP, and ADONIS2, which are widely used in ecological and metagenomic analyses, were employed to compare the signal distribution pattern. The ANOSIM statistic R, MRPP statistic A, and ADONIS2 statistic F were also calculated. Compared with ANOSIM R, ADONIS2 F, and accumulative signal difference, MRPP A had the largest fold changes between TP and FP deletion variations (Figure 4G-H). It is also observed that FP deletion variations had lower MRPP A values than TP deletions (Figure 4I-L). Thus, MRPP could be used in downstream analysis to filter artificial deletion variations.

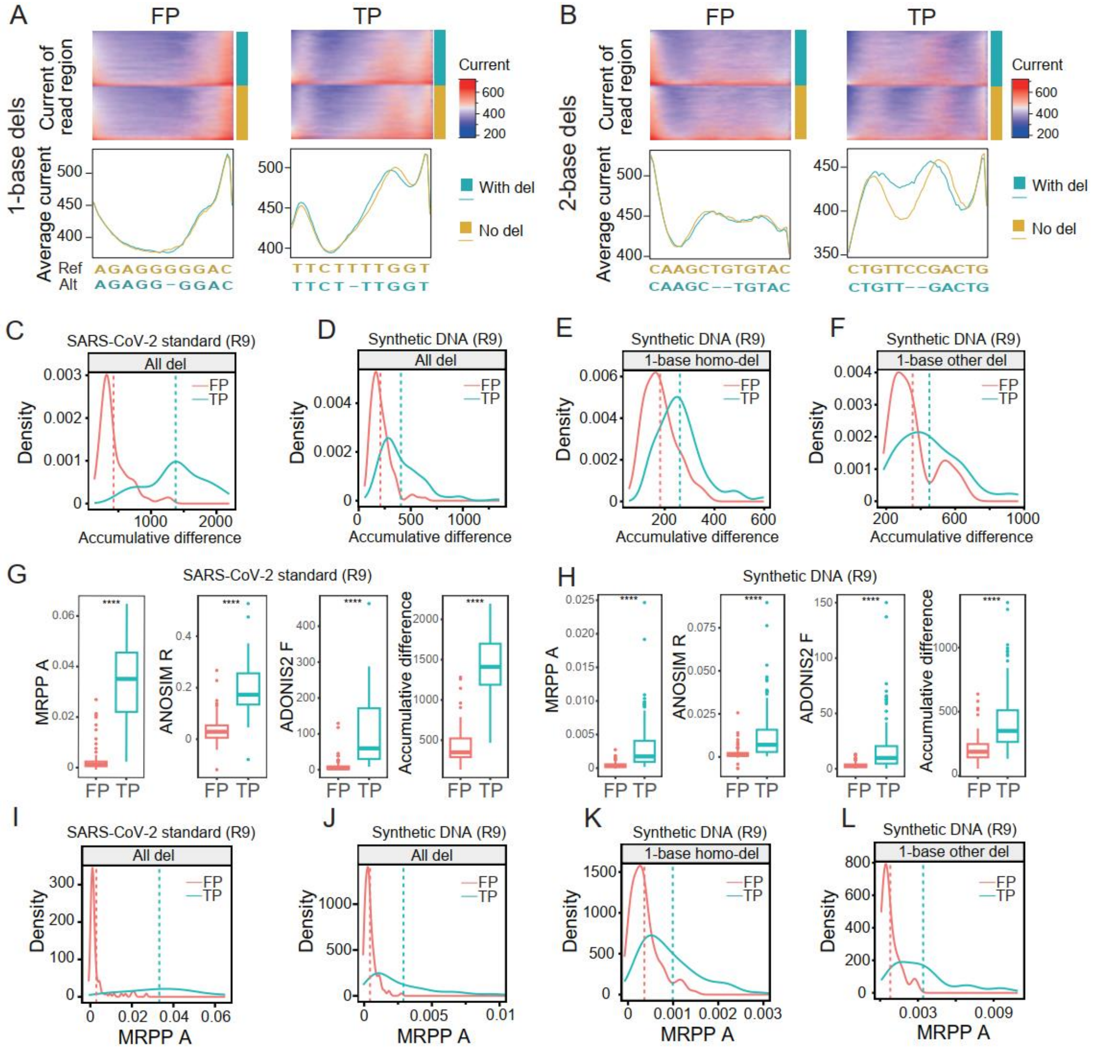

Figure 4. The features of normalized current signals in R9 sequencing data. (A) Heatmaps of normalized current signals from reads with and without 1-base FP and TP homo-del variations. The line plots represented each column's average normalized current measurements in the heatmap. The alternate alleles corresponding to deletions were displayed. (B) Heatmaps of normalized current signals from reads with and without 2-base FP and TP other-del variations. (C) The accumulative difference of average normalized current measurements from FP and TP deletions in R9 WTA sequencing data. For each deletion variation, the accumulative difference equals the sum of the difference values of normalized current measurements between reads with and without deletions. (D-E) The accumulative difference of average normalized current signals corresponding to all deletions (D), 1-base homo-dels (E), and 1-base other-dels (F) in R9 direct sequencing data. The dashed lines represented the mean values. (G-H) The boxplots of MRPP A, ANOSIM R, ADONIS2 F, and accumulative signal difference in FP and TP deletions in R9 WTA sequencing data (G) and R9 direct sequencing data (H). Boxes represent the interquartile range (IQR) between the first and third quartiles (25th and 75th percentiles, respectively). Lines inside denote the median, and whiskers denote the most extreme values within 1.5 times IQR from the first and third quartiles. Outlier values are represented as points. ****P ≤ 0.0001. (I) The density plot of MRPP A corresponding to FP and TP deletions in R9 WTA sequencing data. (J-L) The

density plot of MRPP A corresponding to all deletions (J), 1-base homo-dels (K), and 1-base other-dels (L) in R9 direct sequencing data. The dashed lines represented the mean values.

Performance assessment of MRPP A, Q score and accumulative difference in identifying artificial deletions

The receiver operating characteristic (ROC) curves were employed to assess the effects of three indexes, MRPP A, Q score and accumulative difference, on distinguishing between FP and TP deletions. In WTA sequencing data, the MRPP A obtained the highest area under the ROC curve (AUC) of 0.98 (accuracy: 91.60%) in distinguishing between artificial and true variations (Supplementary Figure S8A). In R9 direct sequencing samples, the MRPP A outperformed the Q score and the accumulative difference in homopolymeric regions, whose AUCs were 0.85, 0.76, and 0.80, respectively (Figure 5A and Supplementary Figure S8B). For the most challenging artificial 1-base homopolymeric deletions (Figure 5B), MRPP A achieved the highest accuracy of 76.73% than the Q score (69.90%) and the sum of difference (71.39%). For other artificial deletions, MRPP A achieved an AUC of 0.92 and an accuracy of 83.41% (Figure 5C). Moreover, MRPP A also had higher AUCs than ANOSIM R and ADONIS2 F (Supplementary Figure S9).

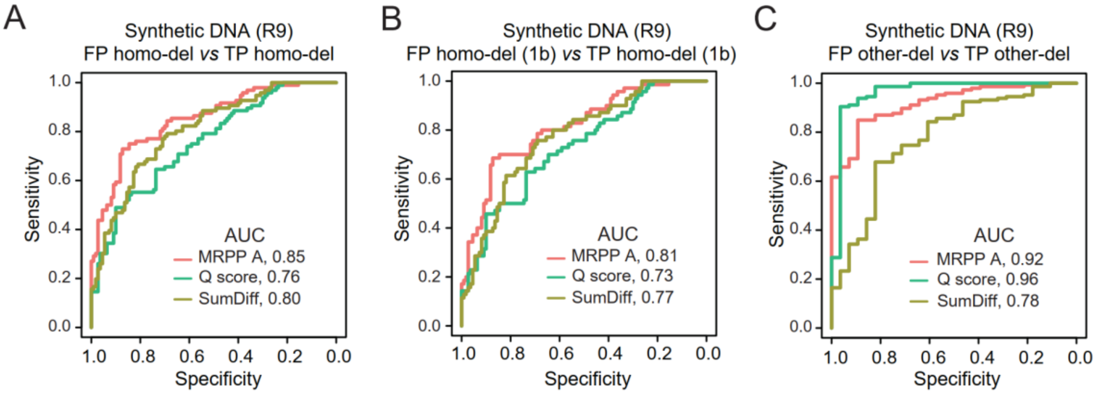

Figure 5. Performance assessment of different indexes in distinguishing between FP and TP deletions in R9 direct sequencing samples. (A-C) The ROCs of MRPP A, Q score, and accumulative difference in distinguishing between FP and TP homo-dels (A), between FP and TP 1-base homo-dels (B), and between FP and TP other-dels (C). 1b: 1-base; SumDiff: sum of difference.

For R10 direct sequencing samples, the Q score was utilized to separate FP from TP deletions under different basecalling models. In the fast basecalling model, Q scores had the weakest performance in distinguishing between FP and TP deletion variations (Supplementary Figure S10), indicating its inapplicability in filtering FP deletion variations. The SUP and HAC models generated fewer FP deletions with higher AUCs (SUP:0.99-1; HAC: 0.89-0.98) (Figure 6, Supplementary Figure S11). For other-dels, the Q score had a higher AUC than homo-dels. Using the Q score alone has achieved a better performance than MRPP A which is used in R9 data (AUC: 0.81-0.92), as revealed by the ROC analyses. Thus, we regard it’s sufficient to employ Q score to filter FP deletion variations for R10. We found average Q scores below 22 could discriminate between FP and TP deletions, which enabled the

identification of 90.63% artificial homo-dels (accuracy: 96.88%) and 100% other-dels (accuracy: 100.00%).

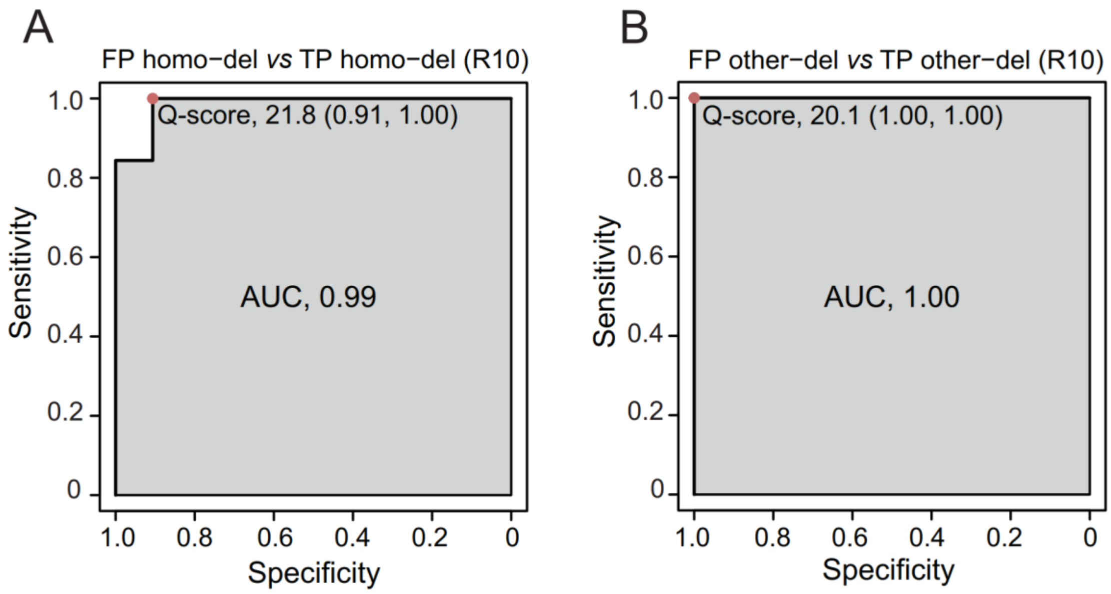

Figure 6. Performance assessment of Q score in distinguishing between FP and TP deletions in R10 direct sequencing samples. (A) The ROC of Q score in distinguishing between FP and TP homo-dels. (B) The ROC of Q score in distinguishing between FP and TP other-dels. The best threshold, specificity, sensitivity, and AUC of the SUP model were plotted.

Implementation of the filtering tool for FP deletions from R9 and R10 nanopore sequencing

The performance of our method was checked under different sequencing depths. MRPP A achieved stable AUCs with strand-specific sequencing depths ≥ 100X in WTA sequencing data (Supplementary Figure S12A). For R9 direct sequencing samples, MRPP A began to outperform the Q score at 400X except in other-del variations (Figure 7A-B, Supplementary Figure S12B-C). Thus, MRPP A or Q score would be utilized to filter FP deletions with varied sequencing depths. The Q score could distinguish between FP and TP variations with strand-specific sequencing depths ≥ 20X in R10 sequencing samples (Supplementary Figure S12D). The corresponding thresholds of MRPP A or Q score were further determined (Table 1). We then developed a tool named Delter to filter artificial deletion variations from R9 or R10 sequencing data, which could choose an appropriate index depending on sequencing protocol, flow cell, and depth. Variations with indexes lower than the recommended thresholds would be predicted as artificial deletions.

We recorded the computational resources for variation calling with LoFreq and deletion filtering with Delter. The runtime and RAM usage required by LoFreq scales with the total bases (Supplementary Figure S13 and Table S7). The computational costs of Delter depend on the flow cell, sequencing protocol, and sequencing depth. Delter needs much less runtime and RAM usage in R10 data than R9 data. We found the runtime (0.06-3.93 s/read) and RAM usage (0.07-3.46 MB/read) of Delter scales with the mean number of reads aligned to

each variation site in each sample (referred as mean read number, Supplementary Figure S14 and Table S8). Moreover, the computational resources of basecalling the current signals were also listed (Supplementary Table S9).

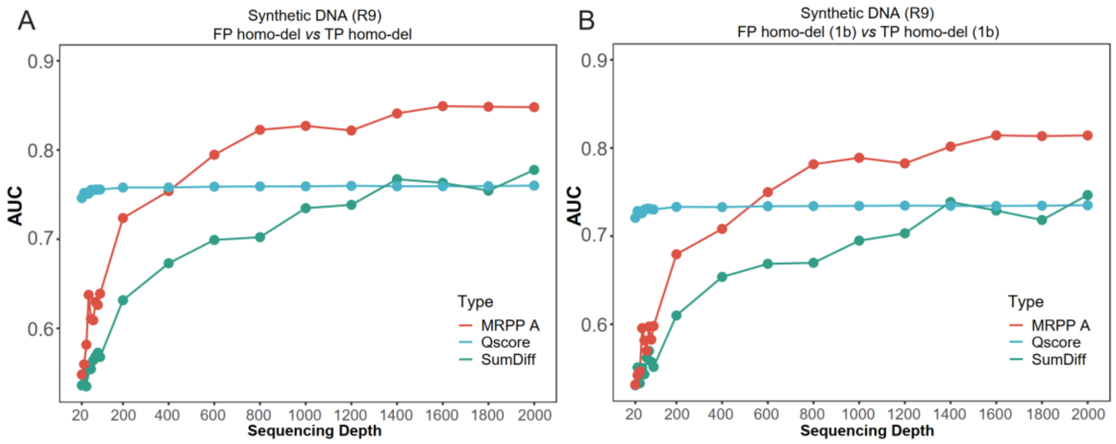

Figure 7. The AUCs distinguishing between TP and FP variations across different sequencing depths. (A-B) The MRPP A- and Q score-derived AUCs corresponding to homo-dels (A) and 1-base homo-dels (B) in R9 direct sequencing data. 1b: 1-base; SumDiff: sum of difference.

Table 1 The suggested index and threshold across different sequencing protocol, flowcell/chemistry, and depth

Flowcell/ chemistry	Sequencing protocol	Sequencing depth*	Index	Threshold
R9	WTA Sequencing	≥20X	MRPP A	0.01
	Direct Sequencing	≥400X	MRPP A	0.001
	Direct Sequencing	[20X,400X)	Q score	23 (Homo-del) 20.6 (Other-del)
R10	Direct Sequencing	≥20X	Q score	21.8 (SUP, Homo-del) 20.0 (SUP, Other-del)

*: strand-specific sequencing depth.

Effective removal of artificial deletions in real samples

Our approach was first validated in 9 samples containing HAdV amplicons (amplicon sequencing) and full-length genes (direct sequencing). In HAdV direct sequencing data, 100% of the true variations (MuAF < 0.15) were correctly identified, and 63 out of 69 artificial homo-dels (MuAF < 0.32) were successfully filtered, achieving an overall accuracy of 91.30% (Table 2). In

HAdV amplicon sequencing data, 4 out of 5 true deletions (MuAF < 0.12) were detected, and 100% of the artificial homo-dels (n=23, MuAF < 0.18) were removed. We further tested our method in a microbial standard sample containing *Veillonella rogosae* (*V. rogosae*), *Bacteroides fragilis* (*B. fragilis*), *Faecalibacterium prausnitzii* (*F. prausnitzii*), *Prevotella corporis* (*P. corporis*), and 5 strains of *E. coli* (B-1109, B-766, B-2207, B-3008, and JM109). After filtering the results using the minimum sequencing depth, the removed FP homo-dels in single bacterial strains (*V. rogosae*, *B. fragilis*, *F. prausnitzii*, and *P. corporis*) ranged from 80.76% to 93.17% (MuAF: 0.06-0.70). In addition, the performance of the Delter workflow in the case of co-existing closely related bacterial strains was evaluated in the ZymoBIOMICS Gut Microbiome Standard. When the *E. coli* B-1109 strain was selected as the reference, the shared deletion variations of the other four strains (B-766, B-2207, B-3008, and JM109) were merged to constitute a list of true deletions (n=236). A total of 130 true deletion variations were recalled (MuAF: 0.10-0.85), with 35 (26.92%) ones misidentified as FP deletions. For the FP deletions (n=14275), 12675 (88.79%, MuAF: 0.08-0.52) were filtered by Delter.

Moreover, three public datasets available on the NCBI SRA database were included for further evaluation. In the ASFV dataset (paired Nanopore R10 and Illumina runs), a total of 76 deletions were retained, six of which were true deletions (MuAF: 0.69-0.93, accuracy=100.00%), with 43 FP deletion variations (43/70=61.43%, MuAF: 0.05-0.28) being filtered, including 25 FP homo-dels. For the *P. aeruginosa* PAO1 dataset (paired Nanopore R10 and Illumina runs), the genome sequence *P. aeruginosa* PAO1 was selected as the reference. Three deletions were identified, including two TP ones (MuAF: 0.21-0.27). The other FP homo-del was successfully filtered by Delter (MuAF=0.33, accuracy=100.00%). In the *B. suis* dataset (paired Nanopore R9 and Illumina runs), 53 deletions were retained. Among these deletions, one true deletion (MuAF=0.61, accuracy=100.00%) was detected. A total of 39 FP deletion variations (39/52=75.00%, MuAF: 0.29-0.54) were filtered, among which 86.37% homo-dels were removed. In summary, these samples proved the efficiency of our filtering method.

Table 2 Validation of the filtering method in real samples

Sample	Deletions/Homo-deletions before filtering		Deletions/Homo-deletions after filtering		Sensitivity	Specificity	Accuracy
	TP	FP	TP	FP			
HAdV full-length gene mixtures (n=4)	5/0	79/69	5/0	10/6	100.00%/-	87.34%/91.30%	88.10%/91.30%
HAdV amplicon mixtures	5/0	27/23	4/0	0/0	80.00%/-	100.00%/100.00%	96.88%/100.00%

(n=5)							
D6331 *E. coli*	130/27	14275/12843	95/12	1600/829	73.08%/44.44%	88.79%/93.55%	88.65%/93.44%
Veillonella rogosae	0/0	5930/5597	0/0	437/382	-/-	92.63%/93.17%	92.63%/93.17%
Bacteroides fragilis	0/0	3535/3477	0/0	509/478	-/-	85.60%/86.25%	85.60%/86.25%
Faecalibacterium prausnitzii	0/0	431/429	0/0	46/46	-/-	89.33%/89.28%	89.33%/89.28%
Prevotella corporis genome 1	0/0	684/660	0/0	131/127	-/-	80.85%/80.76%	80.85%/80.76%
Brucella suis	1/0	52/44	1/0	13/6	100.00%/-	75.00%/86.37%	75.47%/86.37%
African swine fever virus	6/1	70/41	6/1	27/16	100.00%/100.00%	61.43%/60.98%	64.47%/61.90%
Pseudomonas aeruginosa PAO1	2/1	1/1	2/1	0/0	100.00%/100.00%	100.00%/100.00%	100.00%/100.00%

Discussion

Although simplex nanopore sequencing accuracy has increased to Q20+, low-frequency artificial deletion variations still exist in data generated by R9 and R10 flow cells and chemistries, especially in homopolymeric regions. The false positive variations mainly resulted from systematic sequencing errors and are challenging to eliminate. We aim to remove such artificial deletion variations detected at a MuAF threshold of 0.05. The remarkable differences in sequencing signals and Q scores between artificial and true variations were observed. We then developed the first method to filter artificial deletion variations in single samples via current signals or Q scores according to the sequencing protocols, flowcells and depth. Our approach focuses on artificial deletions with MuAF as low as 0.05, and it cannot handle false positive SNVs or insertions of interest at present, which warrants further investigations to unlock its capacity to filter all types of short variations.

We first conducted WTA sequencing using ~5kb SARS-CoV-2 synthetic controls and R9 flow cells. These standard controls are synthesized according to actual SARS-CoV-2 variants. One limitation is that these controls natively lack true deletion variations in homopolymeric regions, while the false deletion

variations are mainly located in homopolymeric regions [23-28]. Trimming bases from both ends of nanopore reads aligned to the reference genome could significantly reduce counts of FP SNVs rather than homo-dels and other-dels, which is consistent with previous studies. The biased distributions of current signals, Q scores, and sequencing speeds between artificial homo-dels and true variations were observed in R9 sequencing data. The high AUC of MRPP A in distinguishing artificial and true variations paved the way for a comprehensive investigation of the characteristics of variations located in homopolymeric regions.

We then synthesized mutant plasmids containing deletion variations in homopolymeric and non-homopolymeric regions relative to wildtype plasmids. We found remarkable differences between reads with and without artificial deletions, with specific preprocessing of raw current signals. As a surrogate of uneven sequencing signals, MRPP A obtained higher AUCs when compared to the Q score and the accumulative sum of normalized signal differences in homopolymeric regions. Notably, the difference is even discernible for the most challenging 1-base homopolymeric deletions, obtaining an AUC > 0.8.

Moreover, we investigated the effects of various sequencing depths on distinguishing between FP and TP variations and found that in some cases (strand-specific sequencing depth < 400X), the Q score should be applied to filter artificial deletions in R9 sequencing data. When sequencing depth is big enough, the MRPP A and sum of difference values outperformed the Q score in homopolymeric regions (Figure 5). However, when the sequencing depth decreased, the Q score was more superior. Therefore, it could be indicated that current signal-related indexes were more sensitive to sequencing depth than the Q score.

For R10 sequencing data, the SUP basecalling model generated the least FP deletion variations than the HAC and fast models. Using the Q score alone has achieved a better performance than MRPP A that is used in R9 data. Average Q scores below 22 could separate FP from TP deletions, filtering > 90.00% artificial homo-dels and other-dels. The Delter workflow was validated in two external datasets composed of paired Illumina and Nanopore R10 sequencing runs of the same sample (Table 2). It achieved a 100% accuracy for real deletion variations and filtered 61.43%-100.00% FP deletions. Moreover, Delter filters R10 FP deletions using Q-scores, without the need to parse the raw sequencing signals. Thus, Delter needs much less runtime and RAM usage in R10 data than R9 data.

Dorado has now been the default basecaller for ONT data. We re-basecalled the R10 data with Dorado SUP model (v4.1.0) and analyzed the VCF files output by LoFreq with Delter. It’s observed that LoFreq called fewer FP deletion variations in Dorado-basecalled data than in Guppy-basecalled data. Notably, the Delter workflow could filter all the FP deletions in Dorado-basecalled data, which means Dorado can benefit from the filtering procedure employed in Delter (Supplementary Table S10).

Available variant callers like Clair3, Medaka, and NanoCaller, as indicated in Hall’s study [15], were also evaluated using the sequencing data of the synthesized RNA and DNA samples (Supplementary Figure S15). Clair3 is a state-of-the-art ONT variant caller. However, it calls germline variations, which

is not explicitly designed for intra-host SNV and indel calling. In the context of low-frequency intra-host variation detection (expected MuAF = 0.1, 0.2, and 0.5), LoFreq outperforms the other three variant callers. It has the highest recalls and F-scores. Moreover, LoFreq has precision comparable to Clair3. It's observed that Clair3 calls much fewer true variations than LoFreq. Therefore, LoFreq is more suitable for intra-host variation calling. In the context of high-frequency or consensus-level intra-host variation detection (mutated allele frequency >= 0.8), LoFreq has recall comparable to Clair3 and Medaka but calls more false variations than Clair3 and Medaka. Moreover, Clair3 required less runtime and RAM usage than LoFreq (Supplementary Table S11). In summary, we selected LoFreq to call variations as it balanced recall and precision in the context of low-frequency intra-host variation detection. Notably, Delter could filter FP homo-dels called by Clair3, which indicates Clair3 can benefit from our filtering procedure (Supplementary Table S12).

Till now, few tools can identify artificial indels in nanopore sequencing. One feasible strategy is integrating the datasets from multiple samples collected across different time points or from different patients, as recently reported by Variabel [26]. However, this tool is not suitable for single samples. We believe that our method can break the limit of sample size and facilitate the filtering of false deletion variations in single samples. This study demonstrated that our method can accurately filter low-frequency artificial variations in microbial nanopore sequencing data. It has potential applicability in studies of tumor heterogeneity. One limitation is the lack of ground truth references for benchmarking such studies. Numerous intra-host variations could always be identified in tumor samples, which need both *in silico* bioinformatic tools and experimental approaches to confirm artificial and true variations.

Conclusions

By nanopore sequencing of synthetic samples with R9 and R10 flow cells and chemistries, we found that artificial short deletion variations were characterized by differences in current signals and Q scores relative to true variations. The MRPP A or Q score could be employed to filter FP deletions in single samples. The filtering method removed a large proportion of artificial homopolymeric deletions in real samples. We hope the method could facilitate the removal of false variations due to nanopore sequencing errors.

Availability of source code and requirements

Project name: Delter
Project home page: https://github.com/nkuyfq/Delter [45]
Workflow Hub: https://doi.org/10.48546/workflowhub.workflow.1205.2 [46]
Operating system(s): Linux
Programming language: Python and Perl
Other requirements: Snakemake (≥ 7.3) and R (≥ 4.2.2)
License: MIT license
RRID: SCR_026376

Data availability

Nanopore raw data are available from the NCBI BioProjects PRJNA1028169,

PRJNA1028529, and PRJNA1140741. The following public sequencing datasets were reused in this study: African swine fever virus (ASFV) dataset: NCBI BioProject PRJNA1096272; *Pseudomonas aeruginosa* PAO1 dataset: EBI Study accession PRJEB51164 - ERR8958864 & ERR9285397; *Brucella suis* dataset: EBI Study accession PRJEB59317 - ERR10828735 & ERR10820713. Source codes and scripts used to filter artificial deletion variations were integrated into the Snakemake workflow and are available in GitHub [45] and Workflow Hub [46] . Demo data for Delter could be accessed via Figshare [47].

Abbreviations

ADONIS2: permutational multivariate analysis of variance; ANOSIM: analysis of similarities; AUC: area under the receiver operating characteristic curve; BAM: Binary Alignment Map; FP: false positive; HAC: high accuracy; HAdV: Human adenovirus; Indel: insertion and deletion; MPS: massive parallel sequencing; MRPP: multi-response permutation procedure; MuAF: mutated allele frequency; ROC: receiver operating characteristic; SMRT: single molecule real-time; SNV: single nucleotide variation; SUP: super accuracy; TP: true positive; Q score: base-quality score; VCF: Variant Call Format; WTA: whole transcriptome amplification.

Declarations

Ethics and consent to participate

N/A

Consent for publication

N/A

Competing Interests

The authors declare that they have no competing interests.

Funding

This research was supported by the Incubation Project of Huadong Research Institute for Medicine and Biotechniques (2024YQFH06).

Authors’ contributions

M.N. designed the experiments and analysis. J.J.Z., T.T.Y., and Z.H.X. designed synthetic sequences, J.R.Z., Y.F.H., and X.H.Y. conducted the sequencing experiments. F.Q.Y., J.J.Z., and X.M.Z. performed the analysis. F.Q.Y. and Z.L.R. built the workflow. M.N. and F.Q.Y. wrote the draft manuscript and revised the manuscript with contributions of other authors. All authors read and approved the final manuscript.

Additional Files

Supplementary Table S1. The information of SARS-CoV-2 synthetic controls.

Supplementary Table S2. The distribution of variations in SARS-CoV-2 synthetic variants against Wuhan-Hu-1 strain.

Supplementary Table S3. The information of synthetic DNA samples.

Supplementary Table S4. The distribution of deletions in mutant plasmids against wildtypes.

Supplementary Table S5. The distribution of deletions in human adenovirus amplicons and full-length genes against references.

Supplementary Table S6. The sequencing summary of synthetic DNA and RNA samples.

Supplementary Table S7. The runtime and RAM usage required by LoFreq.

Supplementary Table S8. The runtime and RAM usage required by Delter.

Supplementary Table S9. The runtime and RAM usage required by basecalling.

Supplementary Table S10. The performance of Delter on Dorado-basecalled R10 data.

Supplementary Table S11. The runtime and RAM usage required by Clair3.

Supplementary Table S12. Validation of the filtering method in deletions called by Clair3.

Supplementary Figure S1. The design of synthetic sequences and mixture samples. (A) The position of deletion variations of the mutated sequences are illustrated as vertical lines. (B) Scheme of mixing the wildtypes and mutants. The mutant and wildtype plasmids were mixed with 1:9, 1:4, and 1:1 ratios for each mixture.

Supplementary Figure S2. The counts of artificial variations before and after trimming bases in different SARS-CoV-2 variants by three variant callers. Only variations with MuAF ≥0.2 were plotted. Trimnum means the number of trimmed bases.

Supplementary Figure S3. The bar plots of four types of artificial variations before (red) and after (blue) trimming bases in R10 direct sequencing samples basecalled with the HAC model (A) and fast model (B). The y-axis was log10 transfromed.

Supplementary Figure S4. The comparisons of Q scores between reads containing deletion variations and reads with no deletions in R10 direct sequencing data basecalled with the HAC model (A) and fast model (B). The dashed lines represented the mean values of Q scores.

Supplementary Figure S5. The comparisons of sequencing speed between reads containing deletion variations and reads with no deletions in R9 WTA sequencing data (A) and R9 direct sequencing data (B) at the scale of the whole read. The dashed lines represented the mean values of speeds. Sequencing speed equals the division of the number of current measurements by the base number.

Supplementary Figure S6. The features of normalized current signals in R9 sequencing data. (A) Heatmaps of normalized current signals from reads with

and without 1-base FP homo-del and 1-base FP other-del variations. The line plots represented each column's average normalized current measurements in the heatmap. The alternate alleles corresponding to deletions were displayed. (B) Heatmaps of normalized current signals from reads with and without 1-base TP homo-del and 1-base TP other-del variations.

Supplementary Figure S7. The accumulative difference of average normalized current measurements from FP and TP deletions stratified by deletion length in R9 WTA sequencing data (A) and R9 direct sequencing data (B). Boxes represent the interquartile range (IQR) between the first and third quartiles (25th and 75th percentiles, respectively). Lines inside denote the median, and whiskers denote the most extreme values within 1.5 times IQR from the first and third quartiles. Outlier values are represented as points. ns: $P > 0.05$; *$P \leq 0.05$; **$P \leq 0.01$; ****$P \leq 0.0001$. 1b: 1-base; 2b: 2-bases; 3b: 3-bases; 6b: 6-bases; 9b: 9-bases.

Supplementary Figure S8. Performance assessment of different indexes in distinguishing between FP and TP deletions in R9 sequencing data. (A) The ROCs of MRPP A, Q score, and accumulative difference in distinguishing between all FP and TP deletions in R9 WTA sequencing data. (B) The ROCs of MRPP A, Q score, and accumulative difference in distinguishing between all FP and TP deletions in R9 direct sequencing data.

Supplementary Figure S9. The AUCs across different bin numbers of MRPP A, ANOSIM R, and ADONIS2 F in distinguishing between FP and TP deletions in R9 WTA sequencing data (A) and R9 direct sequencing data (B).

Supplementary Figure S10. The ROCs and AUCs of Q score in distinguishing between FP and TP deletions in R10 direct sequencing data basecalled with the fast model.

Supplementary Figure S11. Performance assessment of Q score in distinguishing between FP and TP deletions in R10 direct sequencing samples. (A) The ROC of Q score in distinguishing between FP and TP homo-dels. (B) The ROC of Q score in distinguishing between FP and TP other-dels. The best threshold, specificity, sensitivity, and AUC of the HAC model were plotted.

Supplementary Figure S12. The AUCs distinguishing between TP and FP variations across different sequencing depths. (A) The MRPP A- and Q score-derived AUCs corresponding to all deletions at each sequencing depth in WTA sequencing data. (B-C) The MRPP A- and Q score-derived AUCs corresponding to all deletions (B), and other-dels (C) in R9 direct sequencing data. (D) The Q score-derived AUCs corresponding to all deletions, homo-dels, 1-base homo-dels, and other-dels in R10 direct sequencing data. SumDiff: sum of difference.

Supplementary Figure S13. The runtime and RAM usage required by LoFreq. (A) The runtime as a function of the wall-clock time (seconds) divided by the number of total basepairs (Runtime per megabasepairs; y-axis) in each group of samples (x-axis). (B) The runtime as a function of the number of total basepairs in R9 sequencing data. WTA: WTA sequencing of SARS-CoV-2 synthetic RNA controls; Direct: direct sequencing of synthetic DNA plasmids. (C) The runtime as a function of the number of total basepairs in R10 sequencing data of synthetic DNA plasmids that are basecalled with the SUP

or HAC model. (D) The maximum memory usage as a function of the maximum memory usage (MB) divided by the number of total basepairs (Max memory usage per megabasepairs; y-axis) in each group of samples (x-axis). (E) The maximum memory usage as a function of the number of total basepairs in R9 sequencing data. WTA: WTA sequencing of SARS-CoV-2 synthetic RNA controls; Direct: direct sequencing of synthetic DNA plasmids. (F) The maximum memory usage as a function of the number of total basepairs in R10 sequencing data of synthetic DNA plasmids that are basecalled with the SUP or HAC model. Boxes represent the interquartile range (IQR) between the first and third quartiles (25th and 75th percentiles, respectively). Lines inside denote the median, and whiskers denote the most extreme values within 1.5 times IQR from the first and third quartiles. Outlier values are represented as points. ****P ≤ 0.0001. Each point represents a single sample. G1: SARS-CoV-2 synthetic RNA controls sequenced with R9 flow cell, WTA sequencing protocol, and basecalled with the SUP model (R9+WTA seqeuncing+SUP); G2: synthetic DNA plasmids sequenced with R9 flow cell, direct sequencing protocol, and basecalled with the SUP model (R9+Direct sequencing+SUP); G3: synthetic DNA plasmids sequenced with R10 flow cell, direct sequencing protocol, and basecalled with the HAC model (R10+Direct sequencing+HAC); G4: synthetic DNA plasmids sequenced with R10 flow cell, direct sequencing protocol, and basecalled with the SUP model (R10+Direct sequencing+SUP).

Supplementary Figure S14. The runtime and RAM usage required by Delter. (A) The runtime as a function of the wall-clock time (seconds) divided by the mean number of reads aligned to each deletion variation (Runtime per read; y-axis) in each group of samples (x-axis). (B) The runtime as a function of the mean number of reads aligned to each deletion variation in R9 sequencing data. WTA: WTA sequencing of SARS-CoV-2 synthetic RNA controls; Direct: direct sequencing of synthetic DNA plasmids. (C) The runtime as a function of the mean number of reads aligned to each deletion variation in R10 sequencing data of synthetic DNA plasmids that are basecalled with the SUP or HAC model. (D) The maximum memory usage as a function of the maximum memory usage (MB) divided by the mean number of reads aligned to each deletion variation (Max memory usage per read; y-axis) in each group of samples (x-axis). (E) The maximum memory usage as a function of the mean number of reads aligned to each deletion variation in R9 sequencing data. WTA: WTA sequencing of SARS-CoV-2 synthetic RNA controls; Direct: direct sequencing of synthetic DNA plasmids. (F) The maximum memory usage as a function of the mean number of reads aligned to each deletion variation in R10 sequencing data of synthetic DNA plasmids that are basecalled with the SUP or HAC model. Boxes represent the interquartile range (IQR) between the first and third quartiles (25th and 75th percentiles, respectively). Lines inside denote the median, and whiskers denote the most extreme values within 1.5 times IQR from the first and third quartiles. Outlier values are represented as points. ****P ≤ 0.0001. Each point represents a single sample. G1: SARS-CoV-2 synthetic RNA controls sequenced with R9 flow cell, WTA sequencing protocol, and basecalled with the SUP model (R9+WTA seqeuncing+SUP); G2: synthetic DNA plasmids sequenced with R9 flow cell, direct sequencing protocol, and basecalled with the SUP model (R9+Direct sequencing+SUP); G3: synthetic DNA plasmids sequenced with R10 flow cell, direct sequencing protocol, and basecalled with the HAC model (R10+Direct sequencing+HAC); G4: synthetic

DNA plasmids sequenced with R10 flow cell, direct sequencing protocol, and basecalled with the SUP model (R10+Direct sequencing+SUP).

Supplementary Figure S15. Comparison of four variant callers in nanopore sequencing data across different mutated allele frequencies. ns: P > 0.05; **: P ≤ 0.01; ***: P ≤ 0.001; ****: P ≤ 0.0001.

References

1. Li X, Zhang M, Dang C, Wu Z and Xia Y. In situ Nanopore sequencing reveals metabolic characteristics of the Qilian glacier meltwater microbiome. Environ Sci Pollut Res Int 2023;30(35):84805-13. doi:10.1007/s11356-023-28250-0.
2. Quick J, Loman NJ, Duraffour S, Simpson JT, Severi E, Cowley L, et al. Real-time, portable genome sequencing for Ebola surveillance. Nature 2016;530(7589):228-32. doi:10.1038/nature16996.
3. Kafetzopoulou LE, Pullan ST, Lemey P, Suchard MA, Ehichioya DU, Pahlmann M, et al. Metagenomic sequencing at the epicenter of the Nigeria 2018 Lassa fever outbreak. Science 2019;363(6422):74-7. doi:10.1126/science.aau9343.
4. Rhie A, Nurk S, Cechova M, Hoyt SJ, Taylor DJ, Altemose N, et al. The complete sequence of a human Y chromosome. Nature 2023;621(7978):344-54. doi:10.1038/s41586-023-06457-y.
5. Sanderson ND, Kapel N, Rodger G, Webster H, Lipworth S, Street TL, et al. Comparison of R9.4.1/Kit10 and R10/Kit12 Oxford Nanopore flowcells and chemistries in bacterial genome reconstruction. Microb Genom 2023;9(1) doi:10.1099/mgen.0.000910.
6. Zhao W, Zeng W, Pang B, Luo M, Peng Y, Xu J, et al. Oxford nanopore long-read sequencing enables the generation of complete bacterial and plasmid genomes without short-read sequencing. Front Microbiol 2023;14:1179966. doi:10.3389/fmicb.2023.1179966.
7. Chen J, Wang Z, Tan K, Huang W, Shi J, Li T, et al. A complete telomere-to-telomere assembly of the maize genome. Nat Genet 2023;55(7):1221-31. doi:10.1038/s41588-023-01419-6.
8. Sereika M, Kirkegaard RH, Karst SM, Michaelsen TY, Sorensen EA, Wollenberg RD, et al. Oxford Nanopore R10.4 long-read sequencing enables the generation of near-finished bacterial genomes from pure cultures and metagenomes without short-read or reference polishing. Nat Methods 2022;19(7):823-6. doi:10.1038/s41592-022-01539-7.
9. Aganezov S, Yan SM, Soto DC, Kirsche M, Zarate S, Avdeyev P, et al. A complete reference genome improves analysis of human genetic variation. Science 2022;376(6588):eabl3533. doi:10.1126/science.abl3533.
10. Glinos DA, Garborcauskas G, Hoffman P, Ehsan N, Jiang L, Gokden A, et al. Transcriptome variation in human tissues revealed by long-read sequencing. Nature 2022;608(7922):353-9. doi:10.1038/s41586-022-05035-y.
11. Workman RE, Tang AD, Tang PS, Jain M, Tyson JR, Razaghi R, et al. Nanopore native RNA sequencing of a human poly(A) transcriptome. Nat Methods 2019;16(12):1297-305. doi:10.1038/s41592-019-0617-2.
12. Shafin K, Pesout T, Chang PC, Nattestad M, Kolesnikov A, Goel S, et al. Haplotype-aware variant calling with PEPPER-Margin-DeepVariant enables high accuracy in nanopore long-reads. Nat Methods 2021;18(11):1322-32. doi:10.1038/s41592-021-01299-w.
13. Lin JH, Chen LC, Yu SC and Huang YT. LongPhase: an ultra-fast chromosome-scale phasing algorithm for small and large variants. Bioinformatics 2022;38(7):1816-22. doi:10.1093/bioinformatics/btac058.
14. van Dijk EL, Naquin D, Gorrichon K, Jaszczyszyn Y, Ouazahrou R, Thermes C, et al. Genomics in the long-read sequencing era. Trends Genet 2023;39(9):649-71. doi:10.1016/j.tig.2023.04.006.
15. Hall MB, Wick RR, Judd LM, Nguyen AN, Steinig EJ, Xie O, et al. Benchmarking reveals superiority of deep learning variant callers on bacterial nanopore sequence

data. Elife 2024;13 doi:10.7554/eLife.98300.
16. Wang Y, Zhao Y, Bollas A and Au KF. Nanopore sequencing technology, bioinformatics and applications. Nat Biotechnol 2021;39(11):1348-65. doi:10.1038/s41587-021-01108-x.
17. Chen P, Sun Z, Wang J, Liu X, Bai Y, Chen J, et al. Portable nanopore-sequencing technology: Trends in development and applications. Front Microbiol 2023;14:1043967. doi:10.3389/fmicb.2023.1043967.
18. Bull RA, Adikari TN, Ferguson JM, Hammond JM, Stevanovski I, Beukers AG, et al. Analytical validity of nanopore sequencing for rapid SARS-CoV-2 genome analysis. Nat Commun 2020;11(1):6272. doi:10.1038/s41467-020-20075-6.
19. Liu H, Li J, Lin Y, Bo X, Song H, Li K, et al. Assessment of two-pool multiplex long-amplicon nanopore sequencing of SARS-CoV-2. J Med Virol 2022;94(1):327-34. doi:10.1002/jmv.27336.
20. Martignano F, Munagala U, Crucitta S, Mingrino A, Semeraro R, Del Re M, et al. Nanopore sequencing from liquid biopsy: analysis of copy number variations from cell-free DNA of lung cancer patients. Mol Cancer 2021;20(1):32. doi:10.1186/s12943-021-01327-5.
21. Talsania K, Shen TW, Chen X, Jaeger E, Li Z, Chen Z, et al. Structural variant analysis of a cancer reference cell line sample using multiple sequencing technologies. Genome Biol 2022;23(1):255. doi:10.1186/s13059-022-02816-6.
22. Zheng Z, Su J, Chen L, Lee Y-L, Lam T-W and Luo R. ClairS: a deep-learning method for long-read somatic small variant calling. 2023:2023.08.17.553778. doi:10.1101/2023.08.17.553778 %J bioRxiv.
23. Rang FJ, Kloosterman WP and de Ridder J. From squiggle to basepair: computational approaches for improving nanopore sequencing read accuracy. Genome Biol 2018;19(1):90. doi:10.1186/s13059-018-1462-9.
24. Jain M, Koren S, Miga KH, Quick J, Rand AC, Sasani TA, et al. Nanopore sequencing and assembly of a human genome with ultra-long reads. Nat Biotechnol 2018;36(4):338-45. doi:10.1038/nbt.4060.
25. Cretu Stancu M, van Roosmalen MJ, Renkens I, Nieboer MM, Middelkamp S, de Ligt J, et al. Mapping and phasing of structural variation in patient genomes using nanopore sequencing. Nat Commun 2017;8(1):1326. doi:10.1038/s41467-017-01343-4.
26. Liu Y, Kearney J, Mahmoud M, Kille B, Sedlazeck FJ and Treangen TJ. Rescuing low frequency variants within intra-host viral populations directly from Oxford Nanopore sequencing data. Nat Commun 2022;13(1):1321. doi:10.1038/s41467-022-28852-1.
27. Delahaye C and Nicolas J. Sequencing DNA with nanopores: Troubles and biases. PLoS One 2021;16(10):e0257521. doi:10.1371/journal.pone.0257521.
28. Pages-Gallego M and de Ridder J. Comprehensive benchmark and architectural analysis of deep learning models for nanopore sequencing basecalling. Genome Biol 2023;24(1):71. doi:10.1186/s13059-023-02903-2.
29. Ni M, Chen C, Qian J, Xiao HX, Shi WF, Luo Y, et al. Intra-host dynamics of Ebola virus during 2014. Nat Microbiol 2016;1(11):16151. doi:10.1038/nmicrobiol.2016.151.
30. Lythgoe KA, Hall M, Ferretti L, de Cesare M, MacIntyre-Cockett G, Trebes A, et al. SARS-CoV-2 within-host diversity and transmission. Science 2021;372(6539) doi:10.1126/science.abg0821.
31. Ailloud F, Didelot X, Woltemate S, Pfaffinger G, Overmann J, Bader RC, et al. Within-host evolution of Helicobacter pylori shaped by niche-specific adaptation, intragastric migrations and selective sweeps. Nat Commun 2019;10(1):2273. doi:10.1038/s41467-019-10050-1.
32. Vereecke N, Bokma J, Haesebrouck F, Nauwynck H, Boyen F, Pardon B, et al. High quality genome assemblies of Mycoplasma bovis using a taxon-specific Bonito basecaller for MinION and Flongle long-read nanopore sequencing. BMC Bioinformatics 2020;21(1):517. doi:10.1186/s12859-020-03856-0.
33. De Coster W, D'Hert S, Schultz DT, Cruts M and Van Broeckhoven C. NanoPack: visualizing and processing long-read sequencing data. Bioinformatics 2018;34(15):2666-9. doi:10.1093/bioinformatics/bty149.
34. Li H. Minimap2: pairwise alignment for nucleotide sequences. Bioinformatics 2018;34(18):3094-100. doi:10.1093/bioinformatics/bty191.
35. Danecek P, Bonfield JK, Liddle J, Marshall J, Ohan V, Pollard MO, et al. Twelve years of SAMtools and BCFtools. Gigascience 2021;10(2) doi:10.1093/gigascience/giab008.

36. Wilm A, Aw PP, Bertrand D, Yeo GH, Ong SH, Wong CH, et al. LoFreq: a sequence-quality aware, ultra-sensitive variant caller for uncovering cell-population heterogeneity from high-throughput sequencing datasets. Nucleic Acids Res 2012;40(22):11189-201. doi:10.1093/nar/gks918.
37. Koboldt DC, Zhang Q, Larson DE, Shen D, McLellan MD, Lin L, et al. VarScan 2: somatic mutation and copy number alteration discovery in cancer by exome sequencing. Genome Res 2012;22(3):568-76. doi:10.1101/gr.129684.111.
38. Medaka (2023). Medaka (Version 1.7.3) https://github.com/nanoporetech/medaka/releases/download/v1.7.3/medaka-1.7.3.tar.gz.
39. Zheng Z, Li S, Su J, Leung AW, Lam TW and Luo R. Symphonizing pileup and full-alignment for deep learning-based long-read variant calling. Nat Comput Sci 2022;2(12):797-803. doi:10.1038/s43588-022-00387-x.
40. Ahsan MU, Liu Q, Fang L and Wang K. NanoCaller for accurate detection of SNPs and indels in difficult-to-map regions from long-read sequencing by haplotype-aware deep neural networks. Genome Biol 2021;22(1):261. doi:10.1186/s13059-021-02472-2.
41. ont_fast5_api. https://github.com/nanoporetech/ont_fast5_api.
42. R (2022). R (Version 4.2.2) https://cloud.r-project.org/bin/windows/base/old/4.2.2/.
43. ggsci (2018). ggsci (Version 2.9) https://github.com/nanxstats/ggsci/archive/refs/tags/v2.9.tar.gz.
44. Lin HN and Hsu WL. GSAlign: an efficient sequence alignment tool for intra-species genomes. BMC Genomics 2020;21(1):182. doi:10.1186/s12864-020-6569-1.
45. Ye F, Zhu J, Zhang X, Zhang J, Xie Z, Yang T, Han Y, Yang X, Ren Z and Ni M. (2025) ONT Artificial Deletion Filter-Delter (Version 1). [Computer software]. Software Heritage, https://archive.softwareheritage.org/swh:1:snp:79430faf1a78289783766d1714a5de0141647ed5;origin=https://github.com/nkuyfq/Delter.
46. Ye, Q. (2024). ONT Artificial Deletion Filter-Delter. WorkflowHub. https://doi.org/10.48546/WORKFLOWHUB.WORKFLOW.1205.2.
47. Ye, Qiang (2024). Example dataset containing Fast5 and BAM files. figshare. Dataset. https://doi.org/10.6084/m9.figshare.26093869.v5.

 Click here to access/download;Table;Table1-2.docx

Table 1 The suggested index and threshold across different sequencing protocol, flowcell/chemistry, and depth

**Flowcell/ chemistry**	**Sequencing protocol**	**Sequencing depth***	**Index**	**Threshold**
R9	WTA Sequencing	≥20X	MRPP A	0.01
	Direct Sequencing	≥400X	MRPP A	0.001
	Direct Sequencing	[20X,400X)	Q score	23 (Homo-del) 20.6 (Other-del)
R10	Direct Sequencing	≥20X	Q score	21.8 (SUP, Homo-del) 20.0 (SUP, Other-del)

*: strand-specific sequencing depth.

Table 2 Validation of the filtering method in real samples

Sample	Deletions/Homo-deletions before filtering		Deletions/Homo-deletions after filtering		Sensitivity	Specificity	Accuracy
	TP	FP	TP	FP			
HAdV full-length gene mixtures (n=4)	5/0	79/69	5/0	10/6	100.00%/-	87.34%/91.30%	88.10%/91.30%
HAdV amplicon mixtures (n=5)	5/0	27/23	4/0	0/0	80.00%/-	100.00%/100.00%	96.88%/100.00%
D6331 *E. coli*	130/27	14275/12843	95/12	1600/829	73.08%/44.44%	88.79%/93.55%	88.65%/93.44%
Veillonella rogosae	0/0	5930/5597	0/0	437/382	-/-	92.63%/93.17%	92.63%/93.17%
Bacteroides fragilis	0/0	3535/3477	0/0	509/478	-/-	85.60%/86.25%	85.60%/86.25%

Faecalibacterium prausnitzii	0/0	431/429	0/0	46/46	-/-	89.33%/89.28%	89.33%/89.28%
Prevotella corporis genome 1	0/0	684/660	0/0	131/127	-/-	80.85%/80.76%	80.85%/80.76%
Brucella suis	1/0	52/44	1/0	13/6	100.00%/-	75.00%/86.37%	75.47%/86.37%
African swine fever virus	6/1	70/41	6/1	27/16	100.00%/100.00%	61.43%/60.98%	64.47%/61.90%
Pseudomonas aeruginosa PAO1	2/1	1/1	2/1	0/0	100.00%/100.00%	100.00%/100.00%	100.00%/100.00%

Figure 1

Click here to access/download;Figure;Fig1-240705.pdf

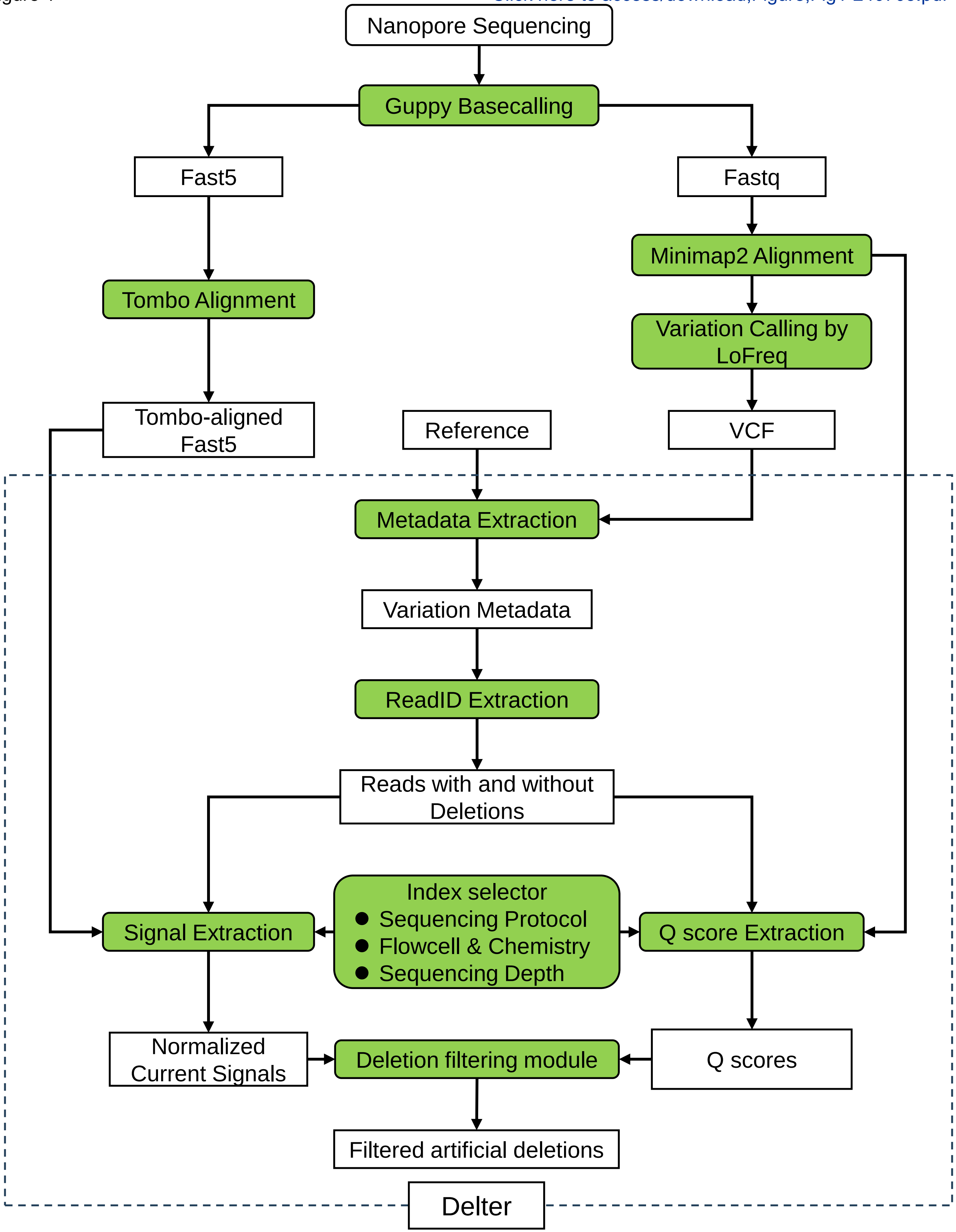

Figure 2

Click here to access/download;Figure;Fig2-241220.pdf

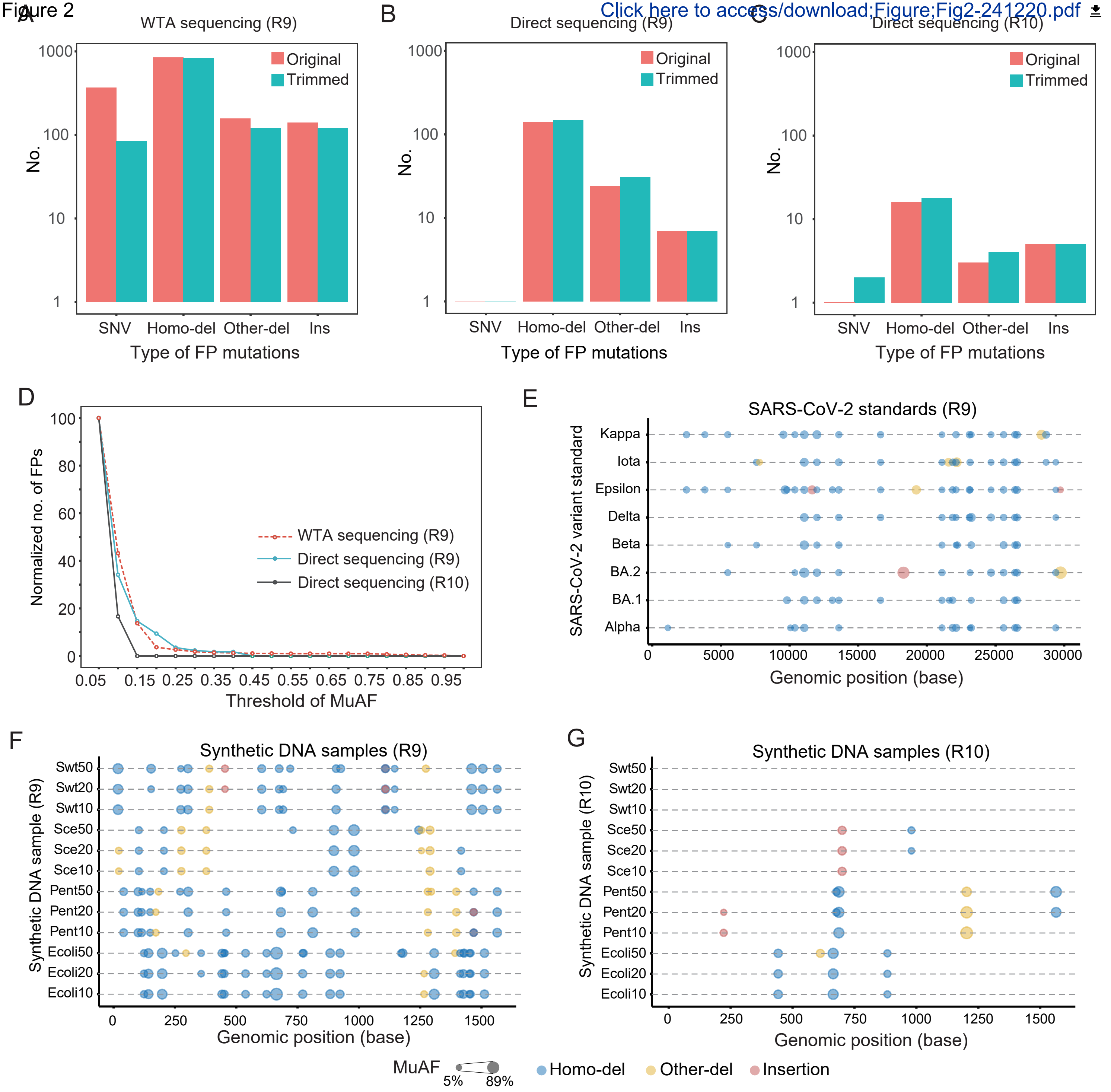

Figure 3

Click here to access/download;Figure;Fig3-241130.pdf

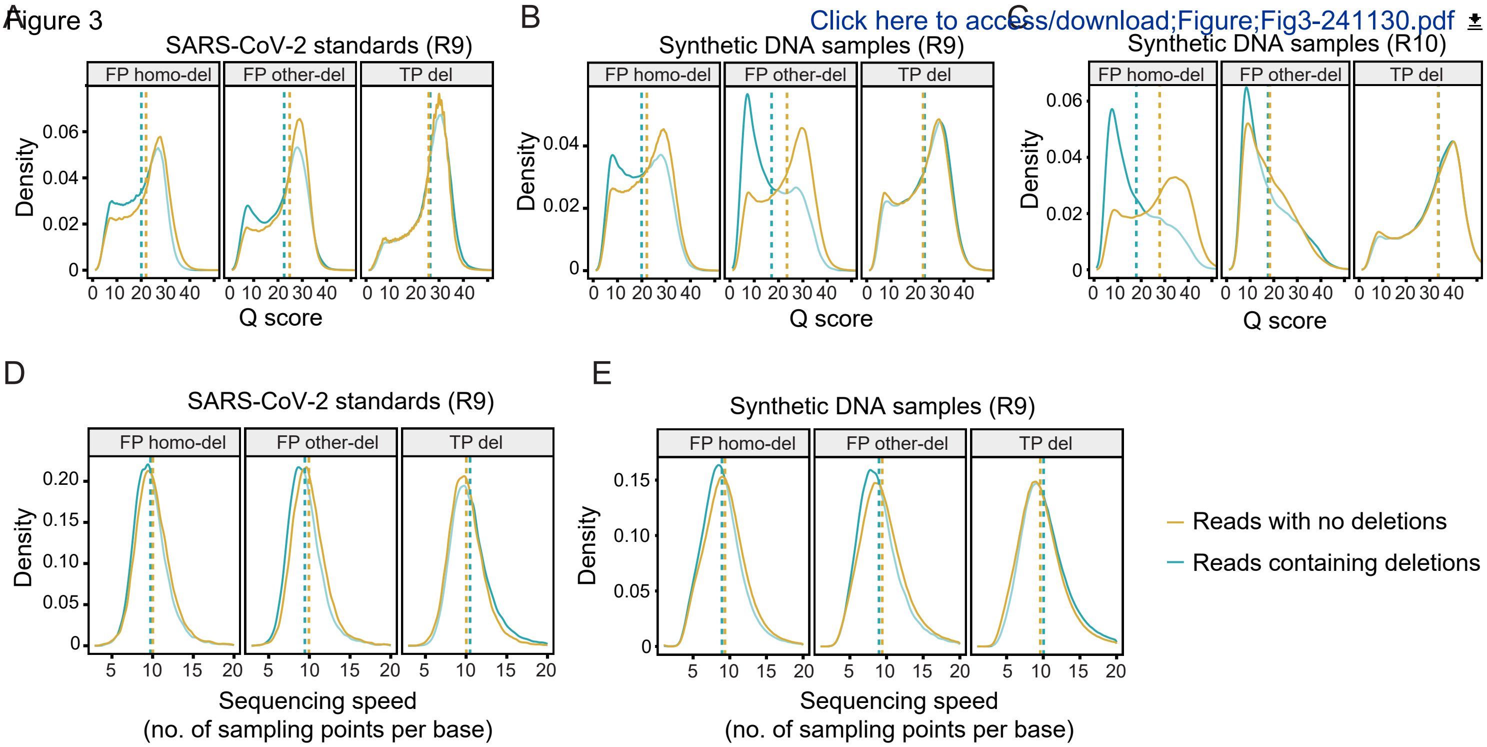

Figure 4

Click here to access/download;Figure;Fig4-241220.pdf

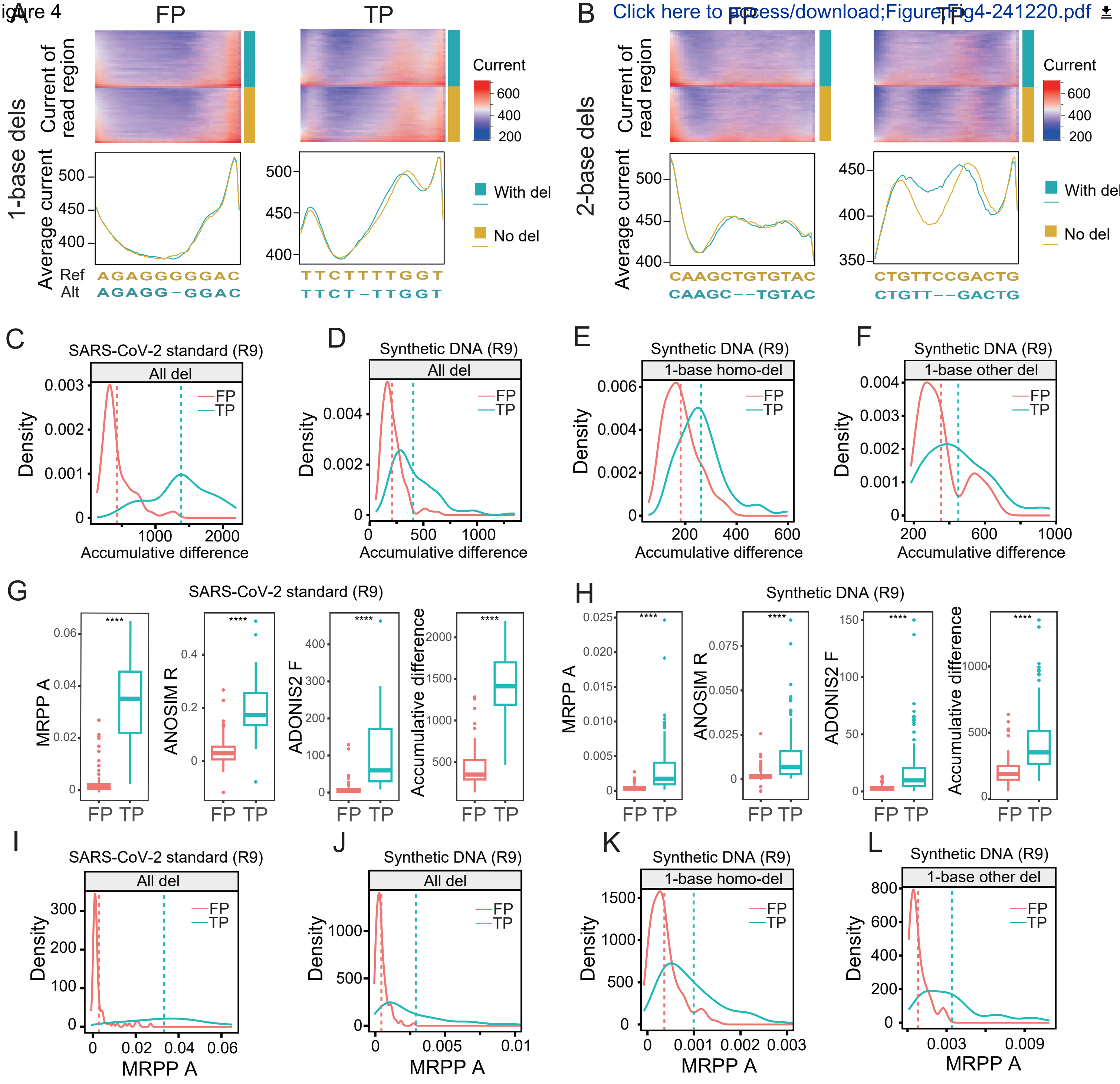

Figure 5

Click here to access/download;Figure;Fig5-240707.pdf

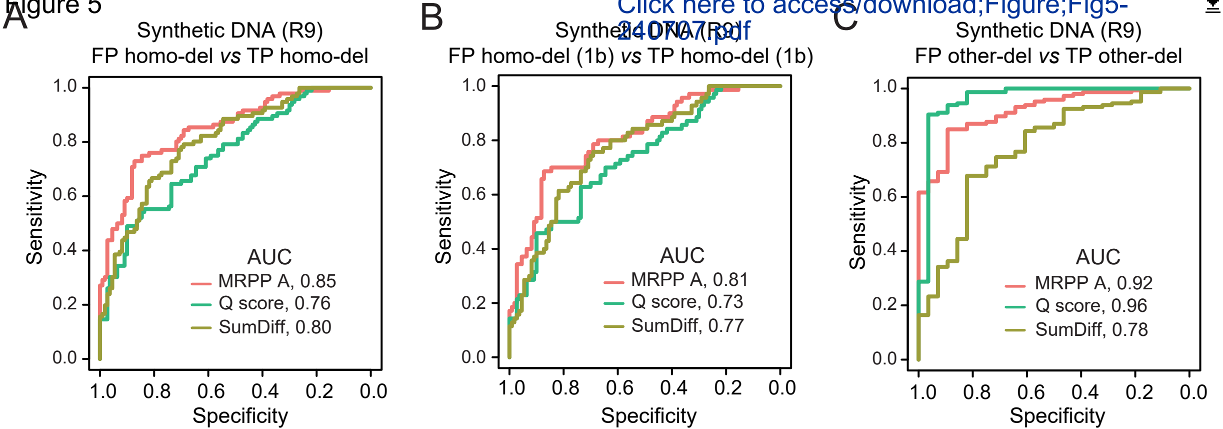

Figure 6

Click here to access/download;Figure;Fig6-

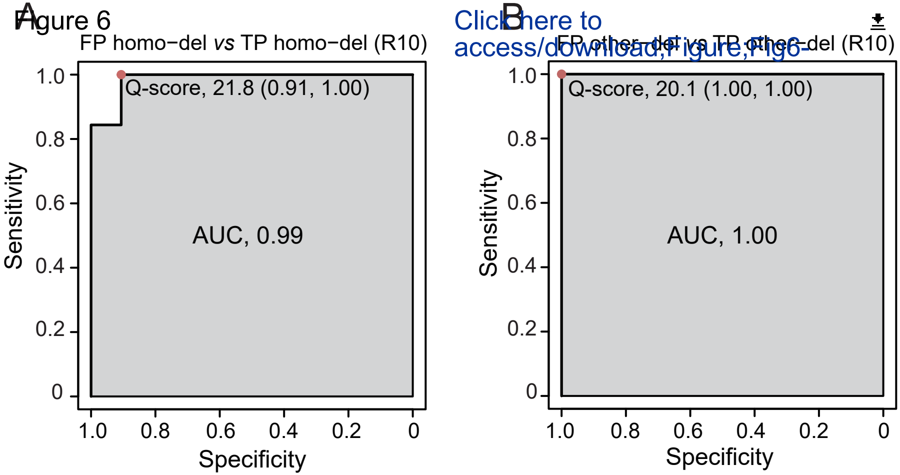

Figure 7

Click here to access/download;Figure;Fig7-241126.pdf

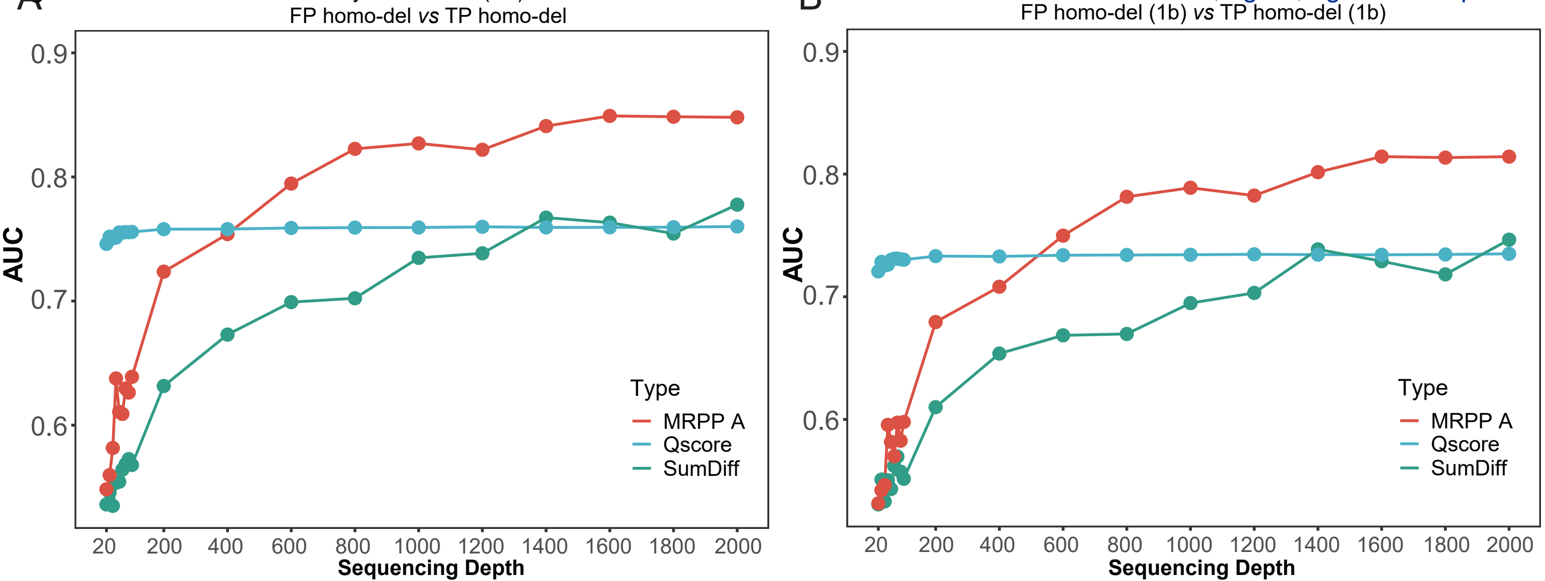

Supplementary Tables

Click here to access/download
Supplementary Material
Supplementary_Table1-12.xlsx

Supplementary Figures 1-15

Click here to access/download
Supplementary Material
Supplementary_Figure1-15.pdf
