# Supplementary material for: Characteristics and filtering of low-frequency artificial short deletion variations based on nanopore sequencing
Source: Gigascience. 2025 Mar 21;14:giaf018. doi: 10.1093/gigascience/giaf018 (PMC11927395; doi:10.1093/gigascience/giaf018)
Supplement: giaf018_Response_to_Reviewer_Comments_Original_Submission [file giaf018_response_to_reviewer_comments_original_submission.pdf]

Author's Response To Reviewer Comments

Responses to the Editor and Reviewers
Editor comments:

Response:
We have added RRIDs in the revised manuscript. The workflow has been registered in workflowhub.eu (https://doi.org/10.48546/workflowhub.workflow.1205.2) and deposited in Github (https://github.com/nkuyfq/Delter).
The point-by-point responses to reviewers have been pasted into the 'Response to Reviewers' box in the submission system. As we cannot paste figures and tables in the 'Response to Reviewers' box, we also uploaded the point-by-point responses to reviewers, embedded with figures and tables, as Supplementary Material in the submission system.

Reviewer comments:

Reviewer #1:
I have placed my comments in the order that they appear in the manuscript.

Response:
Thank you very much for your valuable comments which have significantly improved the manuscript. The point-by-point responses to reviewers have been pasted into the 'Response to Reviewers' box in the submission system. As we cannot paste figures and tables in the 'Response to Reviewers' box, we also uploaded the point-by-point responses to reviewers, embedded with figures and tables, as Supplementary Material in the submission system.

1.The conclusion statement in the abstract is a little vague.

Response:
Thank you for the constructive comment. The conclusion statement has been updated as follows:
Low-frequency artificial short deletion variations, especially the most challenging homopolymeric deletions, could be effectively filtered by Delter using normalized current signals or Q scores according to the employed sequencing strategies.

2.L62-65 portability does indeed distinguish ONT from other sequencing technologies; however, the ability to produce long reads does not distinguish ONT from PacBio.

Response:
Thanks for the valuable comment. The statements have been updated as follows:
Nanopore sequencing is distinguished by its high portability, compared to other commercially available sequencing technologies such as the single molecule real-time (SMRT) sequencing by PacBio (CA, USA) and massive parallel sequencing (MPS) by Illumina (CA, USA) and MGI Tech (Shenzhen, China).

3.L73-74 the reference [4] that supports the statement of ONT producing reads as long as 4Mbp is based on an internal run by ONT as stated on twitter. I would argue this isn't a reliable source to cite as it has not been proven to have been rigorously validated - it is just a statement on twitter. However this is a very minor point.

Response:
Thanks for the valuable comment. The statement has been deleted.

4.L138 Nine is mispelled as Nive

Response:
We are sorry for this stupid mistake. The spelling has been corrected.

5.Table S4 you have labeled any indel with the insertion or deletion of the same nucleotide as being a homopolymer, even if it is just one base. I would argue that this does not constitute a homopolymer, and that a homopolymer is N (4?) or more insertions/deletions of the same base. (find a reference to support this) or was the context surrounding these indels a homopolymer? it isn't clear from the table or the text

Response:
We are sorry for the vague definitions. We focus on short deletions located in homopolymeric regions containing short repeats with ≥ 3 same bases. The "Homo" subtype in Table S4 means that the corresponding deletion occurs in a homopolymeric region. Namely, the context surrounding the deletion is a homopolymer.
In the manuscript, we have defined homo-dels and other-dels around L320: Abundant artificial (false positive, FP) low-frequency variations were identified (Figure 2A-C). Among all the FP types, the small deletions located in ≥ 3-base homopolymeric regions (denoted as homo-dels) were remarkably dominating (62.07%-79.68%), followed by the deletions in the non-homopolymeric regions (other-dels, 10.40%-16.58%).

6.L183-185 it is stated that LoFreq is applicable for analysing nanoproe sequencing data. However, LoFreq was published in 2012, before nanopore was commercialised, and there is no indication in their documentation/paper of applicability to nanopore data. Indeed there is scarce use of this tool on nanopore data in the literature, with one of the only examples I could find having worse performance than other methods (https://www.sciencedirect.com/science/article/pii/S1872497323001059). This is quite a fundamental part of the present study as your determination of indels and thus the error rates is based on the performance of the variant caller used. I would strongly recommend using a more thoroughly tested and validated ONT variant caller such as Clair3 which is designed to work with the latest ONT data. In Figure S2 Medaka and VarScan are compared to LoFreq and it seemed to me that Medaka actually performed better. So again, I am not sure why LoFreq has been selected.

Response:
Thank you for the constructive comment.
Our study focuses on low-frequency intra-host deletion variations, which are similar to somatic variations in human cancers. Clair3 is a state-of-the-art ONT variant caller. However, it's trained with human genome data and designed for calling germline variations, which is unsuitable for our current scenario and not explicitly designed for intra-host SNV and indel calling. LoFreq makes full use of base-call qualities and other sources of errors inherent in sequencing (e.g., mapping or base/indel alignment uncertainty), which are usually ignored by other methods or only used for filtering. LoFreq can run on almost any type of aligned sequencing data since no machine- or sequencing-technology dependent thresholds are used. It automatically adapts to changes in coverage and sequencing quality and can therefore be applied to a variety of datasets, e.g., viral/quasispecies, bacterial, metagenomics or somatic data, which is quite suitable for our current study.
In Bull's study, Varscan2 was used to detect intra-specimen sub-consensus frequencies (mutated allele frequency < 0.8) in nanopore sequencing data of SARS-CoV-2 genomes (PMID: 33298935). In a more recent study focusing on identifying low frequency (< 0.5 allele frequency) intra-host variants directly from multi-sample Oxford Nanopore sequencing data, LoFreq was used to call variations and usually had higher recalls than Clair3 (Figure 3C in PMID: 35288552, Rescuing low frequency variants within intra-host viral populations directly from Oxford Nanopore sequencing data). In this study, the authors reported that more than 70% of the FP calls from Clair3 have allele frequencies less than 0.5 and for ultralow frequency variants (less than 0.1 allele frequency), Clair3 has an FP rate of 100% (all variants identified are false positives).
Available variant callers, as indicated in a recent study (Benchmarking reveals superiority of deep learning variant callers on bacterial nanopore sequence data), were evaluated using our sequencing data. As (1) Longshot does not provide indel calls and (2) DeepVariant supports germline variant-calling in diploid organisms and cannot handle somatic data or any other samples where the genotypes go beyond two copies of DNA, we excluded them and included LoFreq, Clair3, Medaka, and NanoCaller in our comparison based on the sequencing data of the synthesized RNA and DNA samples.
In the context of low-frequency intra-host variation detection (expected mutated allele frequency = 0.1, 0.2, and 0.5), LoFreq outperforms the other three variant callers (Supplementary Figure S15). It has the

highest recalls and F-scores. It's observed that Clair3 calls much fewer true variations than LoFreq. In addition, Clair3 calls zero false variations in most cases, leading to precisions with values of 1 in spite of lower recalls than LoFreq. However, LoFreq has precisions comparable to Clair3. Therefore, LoFreq is more suitable for low-frequency intra-host variation calling.
In the context of high-frequency or consensus-level intra-host variation detection (mutated allele frequency >= 0.8), LoFreq has recalls comparable to Clair3 and Medaka (Supplementary Figure S15). It calls more false variations than Clair3 and Medaka. Thus, Clair3 and Medaka are recommended in such scenarios.
In summary, we selected LoFreq as it balanced recalls and precisions in the context of low-frequency intra-host variation detection.

7.The y-axis scales of Figure 2A-C and Sigure S3 should be the same to allow comparison across plots. In addition, the ordering of the x-axis scale in Figure S3 should be the same as Figure 2A-C.

Response:
Thank you for the valuable comment. We have replaced Figure 2C with the results of R10 SUP data. The y-axis scales were all log10-transformed in order to display for better visualization. The ordering of the x-axis scale in Figure S3 has been changed to be the same as Figure 2A-C.

8.L334-337 it isn't clear to me what you mean when you say HAC was selected for comparable accuracy? Comparable to what? I also don't understand why you focus on investigating the HAC model given the SUP model has less errors? There doesn't seem to be much point in looking at errors in HAC when you can just use SUP basecalling to remove most of the errors. It would be a more useful application to focus on how to remove errors in SUP. In addition to this, if I am interested in calling low-frequency variations, I would use SUP basecalling and not HAC, so again, I don't see the benefit in focusing on HAC.

Response:
Thank you for the valuable comment. In the beginning, given that the HAC model generated more FP deletion variations than the SUP model, we selected the HAC basecalling model for further analyses in order to conduct a comprehensive investigation of the artificial variations. In the latest manuscript, we have used the SUP model to conduct further analyses.

9.L380 it might be worth re-emphasising this is for the fast basecalling model.

Response:
Thank you for the valuable comment. We have rewritten this sentence: Moreover, the differences between reads with and without deletions were negligible in the FP homo-dels derived from R10 direct sequencing samples basecalled with the fast basecalling model (Supplementary Figure S4B).

10.For the section "Performance assessment of MRPP A, Q score and accumulative difference in identifying artificial deletions" I would again prefer to see the focus on SUP rather than HAC. i.e., Figure 6 should be replaced with SUP and the HAC moved to the supplement and L494-497 should describe the accuracy achieveable for SUP with the Q score filtering.

Response:
Thank you for the constructive comment. We have replaced the HAC model with the SUP model in Figure 6 and described the accuracy achievable for SUP with the Q score filtering.

11.What is the significance of the black dashed lines in Figure 7 and S10? Adding a definition in the figure caption would be appreciated.

Response:
Thank you for the valuable comment. The dashed lines in the figure were removed.

12.Apologies if I have missed it, but I didn't see a mention of why MRPP A is not assessed for R10?

Response:
Thank you for the constructive comment. Some statements have been added into the manuscript. When

we started the study in 2022, no tool could be available to align the current signals to the reference sequences for R10 sequencing data. Moreover, using the Q score alone has achieved a better performance (SUP AUC: 0.99-1; HAC AUC: 0.89-0.98) than MRPP A which is used in R9 data (AUC: 0.81-0.92), as revealed by the ROC analyses. Thus, we regard it's sufficient to employ Q score to filter FP variations for R10. Moreover, using the Q score for R10 data requires less computational resources than MRPP A (Supplementary Figure S14 and Table S8).

13.L566-567 a reference is needed to support this

Response:
Thank you for the constructive comment. We have added references.

14.L572-579 this is for R9 data right? Maybe clarify this in the text

Response:
Thank you for the valuable comment. We have modified this sentence as follows: The biased distributions of current signals, Q scores, and sequencing speeds between artificial homo-dels and true variations were observed in R9 sequencing data.

15.There no mention of the results for R10 data in the Discussion. The Discussion makes it sound like there are lots of errors and your filtering got rid of lots of them. Which is true for R9. However, I am left wondering what the ultimate impact is on R10 SUP data. As R9 flowcells are now discontinued, R10 is the more important flowcell to focus on in my opinion. In particular, SUP basecalling provides superior accuracy and is likely what most people will use when attempting to call variants, especially for low frequency situations.

Response:
Thank you for the constructive comment. We validated the filtering method using two external R10 datasets (Table 2 in the manuscript). We have added new statements about R10 data in the Discussion. For R10 sequencing data, the SUP basecalling model generated the least FP deletion variations than the HAC and fast models. Using the Q score alone has achieved a better performance than MRPP A that is used in R9 data. Average Q scores below 22 could separate FP from TP deletions, filtering > 90.00% artificial homo-dels and other-dels. The Delter workflow was validated in two external datasets composed of paired Illumina and Nanopore R10 sequencing runs of the same sample (Table 2). It achieved a 100% accuracy for real deletion variations and filtered 61.43%-100.00% FP deletions. Moreover, Delter filters R10 FP deletions using Q-scores, without the need to parse the raw sequencing signals. Thus, Delter needs much less runtime and RAM usage in R10 data than R9 data.
Dorado has now been the default basecaller for ONT data. We re-basecalled the R10 data with Dorado SUP model (v4.1.0) and analyzed the VCF files output by LoFreq with Delter. It's observed that LoFreq called fewer FP deletion variations in Dorado-basecalled data than in Guppy-basecalled data. Notably, the Delter workflow could filter all the FP deletions in Dorado-basecalled data, which means Dorado can benefit from the filtering procedure employed in Delter (Supplementary Table S10).

16.As mentioned above, given the choice of variant caller (LoFreq), it feels a little like you have created a strawman (i.e., LoFreq is not a modern best practice variant caller, so gives more errors for which to try and filter out). I think the work you have done to identify differences between FP and TP deletions is very important and I would really like to see it applied to variants from a better caller such as Clair3 DeepVariant as these have been shown many times to be the superior variant callers for ONT data.

Response:
Thank you for the constructive comment.
Our study focuses on low-frequency intra-host deletion variations. In the context of low-frequency intra-host variation detection (expected mutated allele frequency = 0.1, 0.2, and 0.5), LoFreq outperforms Clair3 (Supplementary Figure S15). It has the highest recalls and F-scores. Moreover, LoFreq has precisions comparable to Clair3. It's observed that Clair3 calls much fewer true variations than LoFreq. In a previous study about calling intra-host variations from multi-sample Oxford Nanopore sequencing data (PMID: 35288552), the authors reported that more than 70% of the FP calls from Clair3 have allele

frequencies less than 0.5 and for ultralow frequency variants (less than 0.1 allele frequency), Clair3 has an FP rate of 100% (all variants identified are false positives). Therefore, LoFreq is more suitable for intra-host variation calling.
Although Clair3 calls fewer (even zero) true variations than LoFreq, we still use our pipeline to analyze the deletion variations output by Clair3 in our in-house and external datasets (Supplementary Table S12). It's observed that Delter could filter 28.99%-100% FP homo-dels, which proves Clair3 could benefit from the filtering procedure employed in Delter.

17.The FASTQ data has been uploaded, but a Supplementary Table mapping each Run accession to a condition in the paper is needed. i.e., If I want to download the R10 SUP FASTQ for the Synthetic DNA, which accession do I use?

Response:
Thank you for the valuable comment. We have added the NCBI SRA accession numbers in Supplementary Table S6.

18.Given the raw signal data is assessed in this work, the raw signal data (in fast5 or pod5 format) should also be made available if possible.

Response:
Thank you for the valuable comment. We have uploaded the fast5 files to the NCBI SRA database under the NCBI BioProjects PRJNA1028169 and PRJNA1028529.

19.One final comment, though not necessary, if the variant calling is to be redone with Clair3 or DeepVariant, I would also recommend thinking about basecalling the data with Dorado as this has now been the default basecaller for ONT data for a while now.

Response:
Thank you for the constructive comment.
Although the variant calling is not redone with Clair3 or DeepVariant, we re-basecalled the R10 data with Dorado SUP model (v4.1.0) and analyzed the VCF files output by LoFreq with Delter. It's observed that LoFreq called fewer FP deletion variations in Dorado-basecalled data than in Guppy-basecalled data. However, the Delter workflow could filter all the FP deletions in Dorado-basecalled data, which means Dorado can benefit from the filtering procedure employed in Delter (Supplementary Table S10).

Reviewer #2:
Summary: In this manuscript authors propose a bioinformatics pipeline for filtering low-frequency deletions in Nanopore sequencing data. Authors provide extensive validation of the proposed filtering approach on synthetic SARS-CoV-2, HAdV, and several bacterial controls. Sequencing data from the synthetic controls is used to both inform the choice of the filter metrics and parameters, and to validate the efficacy of the proposed pipeline. Given the abundance of short deletions in Nanopore data as supported by this manuscript and prior studies, and the importance of capturing the low frequency variation for complete assessment of the viral and bacterial pathogen diversity, this study addresses an important and relevant question. While the validations presented by the authors are extensive, the current version of manuscript can be improved by incorporating comparisons with a few additional tools commonly employed in variant calling workflows. Overall, I believe that this manuscript is a good fit for the journal after the feedback provided below is addressed in a satisfactory manner.

Response:
Thank you very much for your valuable comments which have significantly improved the manuscript. The point-by-point responses to reviewers have been pasted into the 'Response to Reviewers' box in the submission system. As we cannot paste figures and tables in the 'Response to Reviewers' box, we also uploaded the point-by-point responses to reviewers, embedded with figures and tables, as Supplementary Material in the submission system.

Major comments

(1) For all variant calling (besides some comparisons for SARS-CoV-2 synthetic genomes) only LoFreq was used as the variant calling tool. Recent benchmarking (Hall, 2024) indicates that Clair3 is a strong choice for variant calling from Nanopore data. Furthermore, other methods that aim to improve handling of indel calls in Nanopore data (Dunn, 2023) tend to provide comparisons with Clair3 as the baseline. Thus, it would be good to assess how well Clair3 handles the FP indels on its own, and whether it can benefit from the filtering procedure employed in Delter.

Response:
Thank you for the constructive comment.
Our study focuses on low-frequency intra-host deletion variations, which are similar to somatic variations in human cancers. Clair3 is a state-of-the-art ONT variant caller. However, it’s trained with human genome data and designed for calling germline variations, which is unsuitable for our current scenario and not explicitly designed for intra-host SNV and indel calling. LoFreq makes full use of base-call qualities and other sources of errors inherent in sequencing (e.g., mapping or base/indel alignment uncertainty), which are usually ignored by other methods or only used for filtering. LoFreq can run on almost any type of aligned sequencing data since no machine- or sequencing-technology dependent thresholds are used. It automatically adapts to changes in coverage and sequencing quality and can therefore be applied to a variety of datasets, e.g., viral/quasispecies, bacterial, metagenomics or somatic data, which is quite suitable for our current study.
In Bull’s study, Varscan2 was used to detect intra-specimen sub-consensus frequencies (mutated allele frequency < 0.8) in nanopore sequencing data of SARS-CoV-2 genomes (PMID: 33298935). In a more recent study focusing on identifying low frequency (< 0.5 allele frequency) intra-host variants directly from multi-sample Oxford Nanopore sequencing data, LoFreq was used to call variations and usually had higher recalls than Clair3 (Figure 3C in PMID: 35288552, Rescuing low frequency variants within intra-host viral populations directly from Oxford Nanopore sequencing data). In this study, the authors reported that more than 70% of the FP calls from Clair3 have allele frequencies less than 0.5 and for ultralow frequency variants (less than 0.1 allele frequency), Clair3 has an FP rate of 100% (all variants identified are false positives).
Available variant callers, as indicated in a recent study (Benchmarking reveals superiority of deep learning variant callers on bacterial nanopore sequence data), were evaluated using our sequencing data. As (1) Longshot does not provide indel calls and (2) DeepVariant supports germline variant-calling in diploid organisms and cannot handle somatic data or any other samples where the genotypes go beyond two copies of DNA, we excluded them and included LoFreq, Clair3, Medaka, and NanoCaller in our comparison based on the sequencing data of the synthesized RNA and DNA samples.
In the context of low-frequency intra-host variation detection (expected mutated allele frequency = 0.1, 0.2, and 0.5), LoFreq outperforms the other three variant callers (Supplementary Figure S15). It has the highest recalls and F-scores. It’s observed that Clair3 calls much fewer true variations than LoFreq. In addition, Clair3 calls zero false variations in most cases, leading to precisions with values of 1 in spite of lower recalls than LoFreq. However, LoFreq has precisions comparable to Clair3. Therefore, LoFreq is more suitable for low-frequency intra-host variation calling.
In the context of high-frequency or consensus-level intra-host variation detection (mutated allele frequency >= 0.8), LoFreq has recalls comparable to Clair3 and Medaka (Supplementary Figure S15). It calls more false variations than Clair3 and Medaka. Thus, Clair3 and Medaka are recommended in such scenarios.
In summary, we selected LoFreq as it balanced recalls and precisions in the context of low-frequency intra-host variation detection.
Although Clair3 calls fewer (even zero) true variations than LoFreq, we still use our pipeline to analyze the deletion variations output by Clair3 in our in-house and external datasets (Supplementary Table S12). It’s observed that Delter could filter 28.99%-100% FP homo-dels, which proves Clair3 could benefit from the filtering procedure employed in Delter.

(2) Evaluation on the external data (i.e. data not used to define filtering parameters) is currently limited. In particular, the assumption that all genomes found in the microbial standard D6331 are identical to provided references is reasonable, but might warrant further investigation. A common approach for validating variant calling accuracy is to use a paired dataset, where both long and short reads are available (since the focus of the study is short deletions, Illumina reads should work well), and use short reads to validate long read based indel calls. Providing more extensive comparisons on real data separate from the original study part would be very useful for assessment of tool's applicability across different scenarios.

Response:
Thank you for the constructive comment.
According to the manufacturer's instructions, the microbial standard D6331 is accurately characterized and contains negligible impurity (< 0.01%). It was constructed by pooling cells from pure cultures of microbial strains. The cells from each pure culture were quantified before pooling. After mixing, the microbial composition was confirmed using NGS-based shotgun sequencing. Therefore, we believe that D6331 might contain very little or even no variation relative to the references. Even if real variations exist in the pure cultures of microbial strains (impurity < 0.01%), they might have negligible effects on filtering the low-frequency false variations (minimum mutated allele frequencies = 0.05). The Delter pipeline filtered 80.85%-92.63% FP deletions in D6331 (Table 2), which proved its effectiveness.
In addition to the Nanopore datasets of real samples produced by our lab, we also searched the NCBI SRA database with the keywords "Nanopore R10 and Illumina", and found two available datasets. Deletions detected in the Illumina data are regarded as true deletion variations. The BioProject PRJNA1096272 had one Nanopore R10 run and four paired Illumina MiSeq runs, which sequenced an African swine fever virus-positive blood sample. After quality control with NanoFilt (nanopore data) or fastp (MiSeq data), all the sequencing data were aligned to the reference genome NC_044945.1 with Minimap2 or bwa, and variations were called with LoFreq. After processing by the Delter pipeline, a total of 76 deletions were retained. Among these deletions, six true deletions (accuracy=100.00%) were detected with none misidentified as FP deletions. A total of 43 FP deletion variations (43/70=61.43%) were filtered, including 25 FP homo-dels (Table 2). In addition, the Pseudomonas aeruginosa (P. aeruginosa) PAO1 dataset contains R10 SUP data generated by GridION (SRA accession number: ERR8958864) and paired MiSeq data (ERR9285397). After quality control with NanoFilt (nanopore data) or fastp (MiSeq data, v0.22.0), all the sequencing data were aligned to the reference genome of P. aeruginosa PAO1 (NC_002516.2) and the VCF files were called with LoFreq. Three deletions were retained, two of which were TP deletions. The other FP deletion was filtered by Delter. Thus, Delter identified TP and FP deletions with 100.00% accuracy (Table 2).
Moreover, a Brucella suis sample SAMEA112370833 in the NCBI SRA database has paired Nanopore R9 (SRA accession number: ERR10828735) and Illumina MiSeq (SRA accession number: ERR10820713) sequencing data. Similar to the pre-processing of PRJNA1096272, quality control with NanoFilt (nanopore data) or fastp (MiSeq data) was conducted. All the clean sequencing data were aligned to the reference genome NC_004310.3 with Minimap2 or bwa, and variations were called with LoFreq. After processing by the Delter pipeline, a total of 53 deletions were retained. Among these deletions, one true deletion (accuracy=100.00%) was detected. A total of 39 FP deletion variations (39/52=75.00%) were filtered, among which 86.37% homo-dels were removed (Table 2).

(3) In order to better understand the applicability of the tool for metagenomic scenarios, it can be helpful to see how well the filtering fares in the presence of closely related bacterial strains. The microbial standard D6331 used by the authors contains 5 strains of E. coli. It can be useful to see if variants specific to each strain (when compared to an outgroup choice of an E. coli strain as reference) pass the filter, while the technical FPs do not.

Response:
Thank you for the constructive comment.
The microbial standard D6331 contains 5 strains of E. coli, namely, B-1109, B-766, B-2207, B-3008, and JM109. We randomly selected B-1109 as the reference genome, and then evaluated the similarities between the other four strains and the reference using fastANI. The average nucleotide identities (ANIs) range from 98.46% to 99.52%. DNAdiff and GSAlign were employed to call variations of each strain relative to the reference B-1109 separately. The shared deletion variations reported by both tools were then merged to constitute a list of true deletions (n=236).
The D6331 nanopore sequencing data was aligned to the B-1109 reference genome. The VCF was called with LoFreq and used as input for the Delter workflow. After filtering the results using the minimum sequencing depth, a total of 130 true deletion variations were recalled, with 35 (26.92%) ones misidentified as FP deletions. For the technical FP deletions (n=14275), 12675 (88.79%) were filtered by Delter (Table 2).

(4) Currently the study does not comment on the computational costs of running the pipeline. Given that Nanopore devices are quite portable, it is important to understand whether the proposed pipeline can be

reasonably executed in the field, or whether an individual workstation or a central computing facility is required for post-processing of the data. Furthermore, as the point (1) is addressed it is important to note CPU and GPU times and RAM usage required for running (a) LoFreq, (b) Clair3, and (c) complete Delter pipeline. Finally, reporting on how the pipeline runtime and memory usage scales with the size of the inputs (relevant variables being: total Gbases of read data, number of reads, and reference genome length) would be informative for future users.

Response:
Thank you for the valuable comment. In some cases, the R10 data consumes around 1GB of RAM memory. Thus, the pipeline could reasonably be executed in the field for post-processing of R10 data. In other cases, the Delter pipeline is recommended to be executed in an individual workstation.
As mentioned in the manuscript, the filter takes several files as inputs: (1) the VCF file output by LoFreq; (2) the sorted BAM files storing alignment of nanopore reads to reference sequence; (3) the reference sequence; (4) the directory storing Tombo-resquiggled single-read fast5 files when R9 flow cell and chemistry are employed. Users should also provide the sequencing protocol (amplicon or direct), flowcell/chemistry (R9 or R10), strand-specific sequencing depth for subsampling, the directory storing the final results, and the base number flanking each variation to extract Q scores and current signals.
As indicated by Figure 1 in the manuscript, the complete Delter pipeline does not include basecalling, Tombo alignment (specific for R9 data), Minimap alignment, and variation calling. Thus, these above steps might need pre-processing in high-performance computer which depends on the flow cell, sequencing protocol and basecalling model. In our case, an individual workstation with 256GB RAM and 24GB GPU RAM is enough for these above steps and the complete Delter pipeline.
We recorded the runtime and maximum memory usage for variation calling with LoFreq and Clair3. The runtime and RAM usage required by LoFreq scales with the total bases and depends on the flow cell, sequencing protocol, and basecalling model (Supplementary Figure S13 and Table S7). SARS-CoV-2 synthetic RNA controls that were sequenced with R9 flow cell, WTA sequencing protocol, and basecalled with the SUP model (referred as R9+WTA seqeuncing+SUP) had the lowest runtime per Mbp (mean: 2.88 s/Mbp) and maximum memory usage per Mbp (mean: 7.06 MB/Mbp; Supplementary Figure S13 A and D). The synthetic DNA plasmids sequenced with R9 flow cell, direct sequencing protocol, and basecalled with the SUP model (R9+Direct sequencing+SUP) required intermediate resources (mean: 391.63 s/Mbp; 11.98 MB/Mbp). Moreover, the synthetic DNA plasmids sequenced with R10 flow cell, direct sequencing protocol, and basecalled with the SUP or HAC model (R10+Direct sequencing+SUP or R10+Direct sequencing+HAC) required the most resources (HAC: 483.08 s/Mbp, 37.16 MB/Mbp; SUP: 484.34 s/Mbp, 36.45 MB/Mbp). It's observed that as the total basepairs grew up, the runtime and memory usage also increased (Supplementary Figure S13 B-C and E-F). In contrast, Clair3 called much fewer true and false positive low-frequency intra-host variations than LoFreq, coupled with much less runtimes (0.08-0.19 s/Mbp) and RAM usage (2.57-9.60 MB/Mbp) than LoFreq which also scales with the total bases (Supplementary Table S11). The GPU RAM usage required by the basecaller Guppy was also reported (Supplementary Table S9). A single NVIDIA GPU with 24GB RAM was enough for basecalling the current signals, with a basecalling speed ranging from 1.30 x 10^6 to 4.92 x 10^6 samples per second (STable S9).
The runtime and RAM usage required by the Delter pipeline were further investigated using the Snakemake benchmark rule. The runtime and RAM usage of Delter depends on the flow cell, sequencing protocol, and sequencing depth. Delter filters R9 FP deletions using the current signals when the sequencing depth is high enough or Q score when the sequencing depth is low. Parsing the raw sequencing signals is a time-consuming step as the read number supporting each deletion site (corresponding to the DP field in the VCF file) in each sample grew up. Delter filters R10 FP deletions using the Q score, without the need to parse the raw sequencing signals. Thus, Delter needs much less runtime and RAM usage in R10 data than R9 data. We found the runtime and RAM usage of Delter scales with the mean number of reads aligned to each variation site in each sample (referred as mean read number, Supplementary Figure S14 and Table S8). SARS-CoV-2 synthetic RNA controls that were sequenced with R9 flow cell, WTA sequencing protocol, and basecalled with the SUP model (referred as R9+WTA seqeuncing+SUP) required the most runtime per read (mean: 3.93 s/read) and maximum memory usage per read (mean: 3.46 MB/read; Supplementary Figure S14 A and D). The synthetic DNA plasmids sequenced with R9 flow cell, direct sequencing protocol, and basecalled with the SUP model (R9+Direct sequencing+SUP) required intermediate resources (mean: 2.86 s/read, 1.06 MB/read). Moreover, the synthetic DNA plasmids sequenced with R10 flow cell, direct sequencing protocol, and basecalled with the SUP or HAC model (R10+Direct sequencing+SUP or R10+Direct sequencing+HAC) required the least resources (HAC: 0.06 s/read, 0.07 MB/read; SUP: 0.06

s/read, 0.07 MB/read). It’s observed that as the read number aligned to the deletion variations grew up, the runtime and memory usage also increased except for the memory usage of R10 data which only consumes around 1GB memory (Supplementary Figure S14 B-C and E-F).

Minor comments
(1) P.5 line 138: Nine instead of "Nive"

Response:
We are sorry for this stupid mistake. The spelling has been corrected.

(2) P.12 line 370-371: authors claim "significantly lower Q scores", but neither figures nor text indicate an associated p-value and test for this claim. Either a test needs to be provided, or the statement should not claim significance

Response:
Thank you for the constructive comment. The statement now doesn’t claim significance.

(3) P.14 line 434 (Figure 4): panels A, B appear to be lower resolution and have distorted fonts for x-axis labels; additionally the small fonts and the high number of panels make this figure difficult to parse, it could be advisable to separate it into several independent figures or to move panels A and B to supplementary information

Response:
Thank you for the constructive comment. The panels A and B have high resolutions in the pdf version of Figure 4. The x-axis labels in panels A and B used another font (Courier New) to highlight the differences between the variations and references. The “-” symbol represents missing nucleotide(s) relative to the corresponding reference sequence. We have replaced the font in the latest Figure 4. Moreover, we have moved some sub-figures in panels A and B to the supplementary figure.

(4) P.17 line 520 (Figure 7): x- and y-axis tick label font size is too small to be legible; additionally, it is unclear/unspecified what the dashed lines in the figure represent

Response:
Thank you for the valuable comment. We have modified the font size of tick labels. And the dashed lines in the figure were removed.

(5) Code and repository: GitHub repository has an informative README with clear installation instructions and sample commands for running the tool. However, due to the GPU requirements for the pipeline I was unable to locally validate the tool.

Response:
We are sorry for the vague documentation. The GPU is required by the basecalling step, which is not a necessity of the current Delter pipeline (Figure 1 in the manuscript). However, a GPU is highly recommended for pre-processing (basecalling) of the raw current signals.

References
Hall, Michael B., Ryan R. Wick, Louise M. Judd, An N. Nguyen, Eike J. Steinig, Ouli Xie, Mark Davies, Torsten Seemann, Timothy P. Stinear, and Lachlan Coin. "Benchmarking reveals superiority of deep learning variant callers on bacterial nanopore sequence data." eLife 13 (2024): RP98300.

Dunn, Tim, David Blaauw, Reetuparna Das, and Satish Narayanasamy. "n PoRe: n-polymer realigner for improved pileup-based variant calling." BMC bioinformatics 24, no. 1 (2023): 98.
