# Supplementary material for: Characteristics and filtering of low-frequency artificial short deletion variations based on nanopore sequencing
Source: Gigascience. 2025 Mar 21;14:giaf018. doi: 10.1093/gigascience/giaf018 (PMC11927395; doi:10.1093/gigascience/giaf018)
Supplement: giaf018_Response_to_Reviewer_Comments_Revision_1 [file giaf018_response_to_reviewer_comments_revision_1.pdf]

Author's Response To Reviewer Comments

Responses to the Editor and Reviewers
Editor comments:
Response:
We have carried out some essential formatting revisions suggested by the Editors.

Reviewer comments:
Reviewer #1:
I appreciate the responses to all of my comments and am happy with everything now.
Response:
Thank you very much for your valuable comments which have significantly improved the manuscript.

Reviewer #2:
Overall the manuscript has been strengthened with additional benchmarks, and the contribution made more comprehensive with the reporting of computational requirements of the tools involved. Reviewer feedback has been addressed diligently and with appropriate care for reporoducibility.

A minor comment for lines 704-710: "has recall" (plural not necessary); "as it balanced recall and precision" (plural not necessary); "which indicates Clair3 can benefit" (proves is a strong guarantee that would warrant more extensive exploration of Clair3 + Delter).
Response:
Thank you very much for your valuable comments which have significantly improved the manuscript. We have replaced “has recalls” with “has recall”, and replaced “has precisions” with “has precision”. In addition, “as it balanced recalls and precisions” has been replaced with “as it balanced recall and precision”. The statement “which proves Clair3 could benefit” has also been modified to “which indicates Clair3 can benefit”.
