# Supplementary material for: Characteristics and filtering of low-frequency artificial short deletion variations based on nanopore sequencing
Source: Gigascience. 2025 Mar 21;14:giaf018. doi: 10.1093/gigascience/giaf018 (PMC11927395; doi:10.1093/gigascience/giaf018)
Supplement: giaf018_Reviewer_1_Report_Original_Submission — Michael Benjamin Hall, PhD -- 8/16/2024 [file giaf018_reviewer_1_report_original_submission.pdf]

Reviewer Report

Title: Characteristics and filtering of low-frequency artificial short deletion variations based on nanopore sequencing

Version: Original Submission **Date:** 8/16/2024

Reviewer name: Michael Benjamin Hall, PhD

Reviewer Comments to Author:

I have placed my comments in the order that they appear in the manuscript.1.The conclusion statement in the abstract is a little vague.2.L62-65 portability does indeed distinguish ONT from other sequencing technologies; however, the ability to produce long reads does not distinguish ONT from PacBio.3.L73-74 the reference [4] that supports the statement of ONT producing reads as long as 4Mbp is based on an internal run by ONT as stated on twitter. I would argue this isn't a reliable source to cite as it has not been proven to have been rigorously validated - it is just a statement on twitter. However this is a very minor point.4.L138 Nine is mispelled as Nive5.Table S4 you have labeled any indel with the insertion or deletion of the same nucleotide as being a homopolymer, even if it is just one base. I would argue that this does not constitute a homopolymer, and that a homopolymer is N (4?) or more insertions/deletions of the same base. (find a reference to support this) or was the context surrounding these indels a homopolymer? it isn't clear from the table or the text6.L183-185 it is stated that LoFreq is applicable for analysing nanoproe sequencing data. However, LoFreq was published in 2012, before nanopore was commercialised, and there is no indication in their documentation/paper of applicability to nanopore data. Indeed there is scarce use of this tool on nanopore data in the literature, with one of the only examples I could find having worse performance than other methods (https://www.sciencedirect.com/science/article/pii/S1872497323001059). This is quite a fundamental part of the present study as your determination of indels and thus the error rates is based on the performance of the variant caller used. I would strongly recommend using a more thoroughly tested and validated ONT variant caller such as Clair3 which is designed to work with the latest ONT data. In Figure S2 Medaka and VarScan are compared to LoFreq and it seemed to me that Medaka actually performed better. So again, I am not sure why LoFreq has been selected.7.The y-axis scales of Figure 2A-C and Sigure S3 should be the same to allow comparison across plots. In addition, the ordering of the x-axis scale in Figure S3 should be the same as Figure 2A-C.8.L334-337 it isn't clear to me what you mean when you say HAC was selected for comparable accuracy? Comparable to what? I also don't understand why you focus on investigating the HAC model given the SUP model has less errors? There doesn't seem to be much point in looking at errors in HAC when you can just use SUP basecalling to remove most of the errors. It would be a more useful application to focus on how to remove errors in SUP. In addition to this, if I am interested in calling low-frequency variations, I would use SUP basecalling and not HAC, so again, I don't see the benefit in focusing on HAC.9.L380 it might be worth re-emphasising this is for the fast basecalling model.10.For the section "Performance assessment of MRPP A, Q score and accumulative difference in identifying artificial deletions" I would again prefer to see the focus on SUP rather than HAC. i.e., Figure 6 should be replaced with SUP and the HAC moved to the supplement and

L494-497 should describe the accuracy achieveable for SUP with the Q score filtering.11.What is the significance of the black dashed lines in Figure 7 and S10? Adding a definition in the figure caption would be appreciated.12.Apologies if I have missed it, but I didn't see a mention of why MRPP A is not assessed for R10?13.L566-567 a reference is needed to support this14.L572-579 this is for R9 data right? Maybe clarify this in the text15.There no mention of the results for R10 data in the Discussion. The Discussion makes it sound like there are lots of errors and your filtering got rid of lots of them. Which is true for R9. However, I am left wondering what the ultimate impact is on R10 SUP data. As R9 flowcells are now discontinued, R10 is the more important flowcell to focus on in my opinion. In particular, SUP basecalling provides superior accuracy and is likely what most people will use when attempting to call variants, especially for low frequency situations.16.As mentioned above, given the choice of variant caller (LoFreq), it feels a little like you have created a strawman (i.e., LoFreq is not a modern best practice variant caller, so gives more errors for which to try and filter out). I think the work you have done to identify differences between FP and TP deletions is very important and I would really like to see it applied to variants from a better caller such as Clair3 DeepVariant as these have been shown many times to be the superior variant callers for ONT data.17.The FASTQ data has been uploaded, but a Supplementary Table mapping each Run accession to a condition in the paper is needed. i.e., If I want to download the R10 SUP FASTQ for the Synthetic DNA, which accession do I use?18.Given the raw signal data is assessed in this work, the raw signal data (in fast5 or pod5 format) should also be made available if possible.19.One final comment, though not necessary, if the variant calling is to be redone with Clair3 or DeepVariant, I would also recommend thinking about basecalling the data with Dorado as this has now been the default basecaller for ONT data for a while now.

Level of Interest

Please indicate how interesting you found the manuscript: Choose an item.

Quality of Written English

Please indicate the quality of language in the manuscript: Choose an item.

Declaration of Competing Interests

Please complete a declaration of competing interests, considering the following questions:

- Have you in the past five years received reimbursements, fees, funding, or salary from an organisation that may in any way gain or lose financially from the publication of this manuscript, either now or in the future?
- Do you hold any stocks or shares in an organisation that may in any way gain or lose financially from the publication of this manuscript, either now or in the future?
- Do you hold or are you currently applying for any patents relating to the content of the manuscript?

- Have you received reimbursements, fees, funding, or salary from an organization that holds or has applied for patents relating to the content of the manuscript?
- Do you have any other financial competing interests?
- Do you have any non-financial competing interests in relation to this paper?

If you can answer no to all of the above, write 'I declare that I have no competing interests' below. If your reply is yes to any, please give details below.

I declare that I have no competing interests

I agree to the open peer review policy of the journal. I understand that my name will be included on my report to the authors and, if the manuscript is accepted for publication, my named report including any attachments I upload will be posted on the website along with the authors' responses. I agree for my report to be made available under an Open Access Creative Commons CC-BY license (http://creativecommons.org/licenses/by/4.0/). I understand that any comments which I do not wish to be included in my named report can be included as confidential comments to the editors, which will not be published.

Choose an item.

To further support our reviewers, we have joined with Publons, where you can gain additional credit to further highlight your hard work (see: https://publons.com/journal/530/gigascience). On publication of this paper, your review will be automatically added to Publons, you can then choose whether or not to claim your Publons credit. I understand this statement.

Yes Choose an item.
