# Supplementary material for: Characteristics and filtering of low-frequency artificial short deletion variations based on nanopore sequencing
Source: Gigascience. 2025 Mar 21;14:giaf018. doi: 10.1093/gigascience/giaf018 (PMC11927395; doi:10.1093/gigascience/giaf018)
Supplement: giaf018_Reviewer_2_Report_Original_Submission — Nicolae Sapoval -- 10/14/2024 [file giaf018_reviewer_2_report_original_submission.pdf]

Reviewer Report

Title: Characteristics and filtering of low-frequency artificial short deletion variations based on nanopore sequencing

Version: Original Submission **Date:** 10/14/2024

Reviewer name: Nicolae Sapoval

Reviewer Comments to Author:

Summary: In this manuscript authors propose a bioinformatics pipeline for filtering low-frequency deletions in Nanopore sequencing data. Authors provide extensive validation of the proposed filtering approach on synthetic SARS-CoV-2, HAdV, and several bacterial controls. Sequencing data from the synthetic controls is used to both inform the choice of the filter metrics and parameters, and to validate the efficacy of the proposed pipeline. Given the abundance of short deletions in Nanopore data as supported by this manuscript and prior studies, and the importance of capturing the low frequency variation for complete assessment of the viral and bacterial pathogen diversity, this study addresses an important and relevant question. While the validations presented by the authors are extensive, the current version of manuscript can be improved by incorporating comparisons with a few additional tools commonly employed in variant calling workflows. Overall, I believe that this manuscript is a good fit for the journal after the feedback provided below is addressed in a satisfactory manner.Major comments(1) For all variant calling (besides some comparisons for SARS-CoV-2 synthetic genomes) only LoFreq was used as the variant calling tool. Recent benchmarking (Hall, 2024) indicates that Clair3 is a strong choice for variant calling from Nanopore data. Furthermore, other methods that aim to improve handling of indel calls in Nanopore data (Dunn, 2023) tend to provide comparisons with Clair3 as the baseline. Thus, it would be good to assess how well Clair3 handles the FP indels on its own, and whether it can benefit from the filtering procedure employed in Delter.(2) Evaluation on the external data (i.e. data not used to define filtering parameters) is currently limited. In particular, the assumption that all genomes found in the microbial standard D6331 are identical to provided references is reasonable, but might warrant further investigation. A common approach for validating variant calling accuracy is to use a paired dataset, where both long and short reads are available (since the focus of the study is short deletions, Illumina reads should work well), and use short reads to validate long read based indel calls. Providing more extensive comparisons on real data separate from the original study part would be very useful for assessment of tool's applicability across different scenarios.(3) In order to better understand the applicability of the tool for metagenomic scenarios, it can be helpful to see how well the filtering fares in the presence of closely related bacterial strains. The microbial standard D6331 used by the authors contains 5 strains of E. coli. It can be useful to see if variants specific to each strain (when compared to an outgroup choice of an E. coli strain as reference) pass the filter, while the technical FPs do not.(4) Currently the study does not comment on the computational costs of running the pipeline. Given that Nanopore devices are quite portable, it is important to understand whether the proposed pipeline can be reasonably executed in the field, or whether an individual workstation or a central computing facility is required for post-processing of the data. Furthermore, as the point (1) is addressed it is important to

note CPU and GPU times and RAM usage required for running (a) LoFreq, (b) Clair3, and (c) complete Delter pipeline. Finally, reporting on how the pipeline runtime and memory usage scales with the size of the inputs (relevant variables being: total Gbases of read data, number of reads, and reference genome length) would be informative for future users.Minor commentsP.5 line 138: Nine instead of "Nive"P.12 line 370-371: authors claim "significantly lower Q scores", but neither figures nor text indicate an associated p-value and test for this claim. Either a test needs to be provided, or the statement should not claim significanceP.14 line 434 (Figure 4): panels A, B appear to be lower resolution and have distorted fonts for x-axis labels; additionally the small fonts and the high number of panels make this figure difficult to parse, it could be advisable to separate it into several independent figures or to move panels A and B to supplementary informationP.17 line 520 (Figure 7): x- and y-axis tick label font size is too small to be legible; additionally, it is unclear/unspecified what the dashed lines in the figure representCode and repository: GitHub repository has an informative README with clear installation instructions and sample commands for running the tool. However, due to the GPU requirements for the pipeline I was unable to locally validate the tool.ReferencesHall, Michael B., Ryan R. Wick, Louise M. Judd, An N. Nguyen, Eike J. Steinig, Ouli Xie, Mark Davies, Torsten Seemann, Timothy P. Stinear, and Lachlan Coin. "Benchmarking reveals superiority of deep learning variant callers on bacterial nanopore sequence data." eLife 13 (2024): RP98300.Dunn, Tim, David Blaauw, Reetuparna Das, and Satish Narayanasamy. "n PoRe: n-polymer realigner for improved pileup-based variant calling." BMC bioinformatics 24, no. 1 (2023): 98.

Level of Interest

Please indicate how interesting you found the manuscript: Choose an item.

Quality of Written English

Please indicate the quality of language in the manuscript: Choose an item.

Declaration of Competing Interests

Please complete a declaration of competing interests, considering the following questions:

- Have you in the past five years received reimbursements, fees, funding, or salary from an organisation that may in any way gain or lose financially from the publication of this manuscript, either now or in the future?
- Do you hold any stocks or shares in an organisation that may in any way gain or lose financially from the publication of this manuscript, either now or in the future?
- Do you hold or are you currently applying for any patents relating to the content of the manuscript?
- Have you received reimbursements, fees, funding, or salary from an organization that holds or has applied for patents relating to the content of the manuscript?
- Do you have any other financial competing interests?

- Do you have any non-financial competing interests in relation to this paper?

If you can answer no to all of the above, write 'I declare that I have no competing interests' below. If your reply is yes to any, please give details below.

I declare that I have no competing interests.

I agree to the open peer review policy of the journal. I understand that my name will be included on my report to the authors and, if the manuscript is accepted for publication, my named report including any attachments I upload will be posted on the website along with the authors' responses. I agree for my report to be made available under an Open Access Creative Commons CC-BY license (http://creativecommons.org/licenses/by/4.0/). I understand that any comments which I do not wish to be included in my named report can be included as confidential comments to the editors, which will not be published.

Choose an item.

To further support our reviewers, we have joined with Publons, where you can gain additional credit to further highlight your hard work (see: https://publons.com/journal/530/gigascience). On publication of this paper, your review will be automatically added to Publons, you can then choose whether or not to claim your Publons credit. I understand this statement.

Yes Choose an item.
